# Eliminating single points of trust: a hybrid quantum and post-quantum blockchain with distributed key generation

**DOI:** 10.1038/s41598-025-23310-6

**Published:** 2025-12-26

**Authors:** Khang Wen Goh, Burhan Ul Islam Khan, Abdul Raouf Khan, Dwi Sudarno Putra, Fathimathul Rajeena P. P., Md. Alamin Bhuyian

**Affiliations:** 1 Faculty of Data Science and Information Technology, INTI International University, 71800 Nilai, Malaysia; 2 Department of Computer System and Technology, Faculty of Computer Science and Information Technology, Universiti Malaya, 50603 Kuala Lumpur, Malaysia; 3 Fakultas Teknik, Universitas Negeri Padang, Padang, 25132 Indonesia; 4 Department of Computer Sciences, King Faisal University, 31982 Hofuf, Al-Ahsa Saudi Arabia; 5 Department of Computer Engineering, King Faisal University, 31982 Hofuf, Al-Ahsa Saudi Arabia

**Keywords:** Quantum blockchain, Post-quantum cryptography (PQC), Distributed key generation (DKG), Delegated proof-of-stake (DPoS), Threshold cryptography, Fully flipped permutation (FFP), Borda count, Mathematics and computing, Physics

## Abstract

Blockchain systems built on classical cryptography face immediate risks from large-scale quantum computers, while purely quantum-based blockchains often rely on a single Private Key Generator (PKG) and incur heavy resource overheads. To overcome these issues, this paper proposes a hybrid quantum and post-quantum blockchain approach that removes single points of trust by using Distributed Key Generation and a dual-layer signature mechanism. This method integrates quantum digital signatures, rooted in the Fully Flipped Permutation problem, with classical post-quantum (lattice-based) cryptography, enabling users to switch between quantum and classical signatures according to security requirements and channel conditions. Delegated Proof-of-Stake with node behavior and Borda count has been incorporated to manage consensus, ensuring that witness nodes are regularly re-elected and malicious actors are penalized by distributing secret shares among multiple rotating witnesses. We eliminate the central vulnerability of a sole PKG while maintaining rigorous resistance to collusions. Our analytical model indicates that a fraction of transactions can use quantum signatures without system-wide bottlenecks, while the remaining transactions follow classical PQC paths with throughput approaching classical baselines under our modeling assumptions. Consequently, this hybrid method offers higher scalability, robust collusion resistance, and long-term security even under quantum-capable adversaries. This paper presents extensive theoretical analyses, probability models, and algorithmic complexities, demonstrating that our design provides resilient infrastructure that meets the key performance and security requirements of next-generation blockchain systems.

## Introduction and background

Blockchain technology has flourished over the last decade, primarily due to its ability to guarantee decentralized, tamper-resistant, and transparent record-keeping in domains ranging from digital currencies to supply chains and beyond^1,2^. However, the foreseeable advent of large-scale quantum computers poses a serious threat to traditional cryptographic primitives, such as those underpinning widely used blockchain schemes^3^. Simultaneously, certain blockchain implementations retain single points of trust or failure, whether through centralized certificate authorities or private key management schemes, thus limiting their professed decentralization and weakening overall security^4^.

In their seminal work, Wang et al.^5^ proposed a quantum blockchain architecture that integrates asymmetric quantum encryption with a Delegated Proof-of-Stake (DPoS) consensus variant, specifically Delegated Proof-of-Stake with node behavior and Borda count (DPoSB). While their approach provides resistance to quantum attacks on classical cryptographic methods, it nonetheless relies on a single Private Key Generator (PKG) during the key distribution and transaction-signing process. This structural reliance introduces a single point of trust and partially defeats the decentralized premise central to blockchain systems. Furthermore, the purely quantum-based signature process, although theoretically sound, may face substantial practical constraints in near-term deployments, such as maintaining stable quantum channels and mitigating quantum decoherence at scale^6^. These limitations highlight the need for a hybrid solution that can both distribute trust and leverage more mature post-quantum (classical) cryptographic methods.

### Motivation

Blockchain systems are widely hailed as “trustless” environments because no central authority controls the ledger^1^. Yet the method of key generation and distribution can create single-trust bottlenecks if entrusted to a single entity. As detailed in “Identified shortcomings” section, the single PKG represents a critical vulnerability that could undermine the entire network’s security. Additionally, relying exclusively on quantum signatures can become resource-intensive and less feasible in mixed classical-quantum network environments. This dilemma prompts two key questions:

- How can blockchains maintain quantum resistance without relying on a single PKG?
- Can a hybrid approach, combining quantum and classical post-quantum methods, balance security, practicality, and efficiency?

In the base quantum blockchain proposal by Wang et al.^5^, transactions are signed using an asymmetric quantum encryption scheme based on the difficulty of Fully Flipped Permutation (FFP) problems. Although this scheme addresses quantum computing threats, it still relies on a central, trusted node to issue and manage private keys. Moreover, quantum-only transaction signing may be overkill for routine transactions, many of which could be protected effectively by classical PQC^7,8^. Consequently, there is a pressing need for a blockchain system that both eliminates single points of trust and supports multiple cryptographic layers, some of which are purely quantum, while others are based on established post-quantum methods, enabling flexible and scalable deployment.

### Related work

The intersection of quantum computing and blockchain technology has sparked significant research across multiple domains. To contextualize our hybrid approach, we examine three primary research directions that have emerged in response to quantum threats: quantum-based blockchain solutions that leverage quantum mechanics for security, classical post-quantum cryptographic schemes that maintain traditional communication protocols while ensuring quantum resistance, and consensus algorithms that must adapt to these new cryptographic paradigms. With IBM’s 433-qubit Osprey processor^9^ and Google’s error-corrected logical qubit demonstrations^10^, the timeline for cryptographically relevant quantum computers continues to accelerate, necessitating the immediate adoption of quantum-resistant protocols. Recent developments in quantum network infrastructure, such as the successful demonstration of intercontinental quantum communication via satellite^11^, further emphasize the urgency of quantum-resistant blockchain solutions. Each approach offers distinct advantages and limitations that inform our design decisions.

#### Quantum-based blockchains

Quantum-secured blockchains have garnered considerable attention due to their potential to resist quantum adversaries^5,12,13^. Common efforts include quantum key distribution (QKD)-backed methods, quantum digital signatures, and block generation protocols that leverage quantum entanglement. Despite their promise, many proposed designs (1) still depend on partially trusted nodes or (2) underestimate the cost and complexity of wide-scale quantum communication infrastructure^12^.

#### Classical post-quantum schemes

In parallel, classical PQC has advanced rapidly, with lattice-based, code-based, and hash-based cryptographic schemes being standardized by bodies such as the National Institute of Standards and Technology (NIST)^7,8^. These PQC methods retain classical data-transmission protocols but rely on mathematically hard problems believed to withstand quantum attacks, such as Ring-Learning with Errors (Ring-LWE) or Multivariate Polynomial problems. Notably, PQC does not require quantum channels; therefore, it is often viewed as a more near-term replacement for Rivest-Shamir-Adleman (RSA) or Elliptic Curve Cryptography (ECC)^2^. The recent NIST standardization of CRYSTALS-Kyber for key encapsulation and CRYSTALS-Dilithium for digital signatures^14^ marks a significant milestone in the practical deployment of PQC.

#### Existing consensus algorithms

Consensus mechanisms like Proof-of-Work (PoW)^1^, Proof-of-Stake (PoS)^2^, and delegated variants such as DPoS^15^ or DPoSB^5^ form the backbone of blockchain. Their goal is to prevent double-spending and ensure that all nodes reach a consensus on the ledger state. However, mining-based approaches can be highly resource-intensive, while stake-based methods must carefully handle malicious or concentrated stakeholders. Wang et al.^5^ demonstrated a stake vote approach combined with the Borda count to punish misbehaving nodes. Despite reducing computational overhead, the DPoSB mechanism retains a single PKG for key management, thereby limiting the benefits of decentralization. Recent hybrid consensus mechanisms, such as Algorand’s pure proof-of-stake^16^ and Ethereum 2.0’s Casper FFG^17^, demonstrate evolving approaches to scalability; however, neither comprehensively addresses post-quantum security.

#### Trust management in distributed systems

Beyond blockchain applications, distributed trust management has been explored in other domains with distinct challenges. In vehicular networks, reputation schemes address mobility-specific concerns such as distinguishing leader–follower roles in platoons (PPDR)^18^, context-aware trust evaluation using reinforcement learning (TROVE)^19^, and cloud-assisted reputation updating with classical cryptography (PPRU)^20^. While these works demonstrate the importance of trust management in distributed systems, they operate under fundamentally different assumptions, dynamic topology, geographic mobility, and ephemeral connections, compared to our static witness-based blockchain framework with quantum cryptographic threats. Our focus remains on threshold-based key management and quantum-resistant signatures rather than mobility or location-based trust challenges.

#### Recent surveys and quantum network constraints

A recent survey contrasts post-quantum and quantum blockchains, highlighting a practical gap: fully quantum schemes face infrastructure immaturity, while classical-only approaches may not guarantee long-term quantum resistance^21^. A theoretical analysis proposes and studies how quantum primitives could support decentralized operation, while noting current limitations and required infrastructures^22^. Meanwhile, quantum-network studies emphasize real-world constraints, probabilistic entanglement generation, finite coherence times, and routing/coordination overheads, which complicate immediate end-to-end deployment^23–25^. Our hybrid design addresses this gap by combining DKG-based distributed trust with a dual signature stack (quantum + PQC): classical throughput by default, with quantum paths opportunistically used where channels exist.

### Problem statement and contributions

While various quantum and post-quantum approaches offer partial solutions, critical gaps remain, particularly in terms of centralized trust and deployment feasibility in mixed network settings. To address these limitations and build on the findings reviewed above, we now define the core problem and outline the contributions of our proposed solution.

#### Problem statement

Existing quantum blockchain solutions, including the one advanced by Wang et al.^5^, address quantum threats but still suffer from the central trust vulnerability detailed in Section “Identified shortcomings”. This reliance on a single PKG or trusted node potentially negates key blockchain tenets of decentralization and censorship resistance while also imposing purely quantum channels on every transaction, a requirement that can be prohibitively expensive or unworkable for near-term production environments.

#### Contributions

To overcome these concerns, this paper introduces a hybrid quantum and post-quantum blockchain architecture that eliminates single points of trust by employing:

- Distributed Key Generation (DKG): We integrate threshold cryptography^26^ into the quantum blockchain to ensure that no single node can unilaterally control or distribute private keys. This shift disperses trust across multiple nodes, mitigating the single PKG’s vulnerability.
- Hybrid Cryptographic Model: While quantum signatures remain an option for high-security or specialized transactions, we also incorporate classical post-quantum signatures (e.g., lattice-based) to reduce reliance on quantum channels. This dual-layer design empowers participants to select an appropriate signing mechanism, balancing security, resource demands, and network conditions.
- Enhanced Consensus Integration: We outline how our DKG and hybrid cryptography can be seamlessly integrated into the DPoSB consensus framework^5^, thereby offering robust defenses against both quantum-based and stake-based attacks while preserving low computational overhead.

**Fig. 1:**
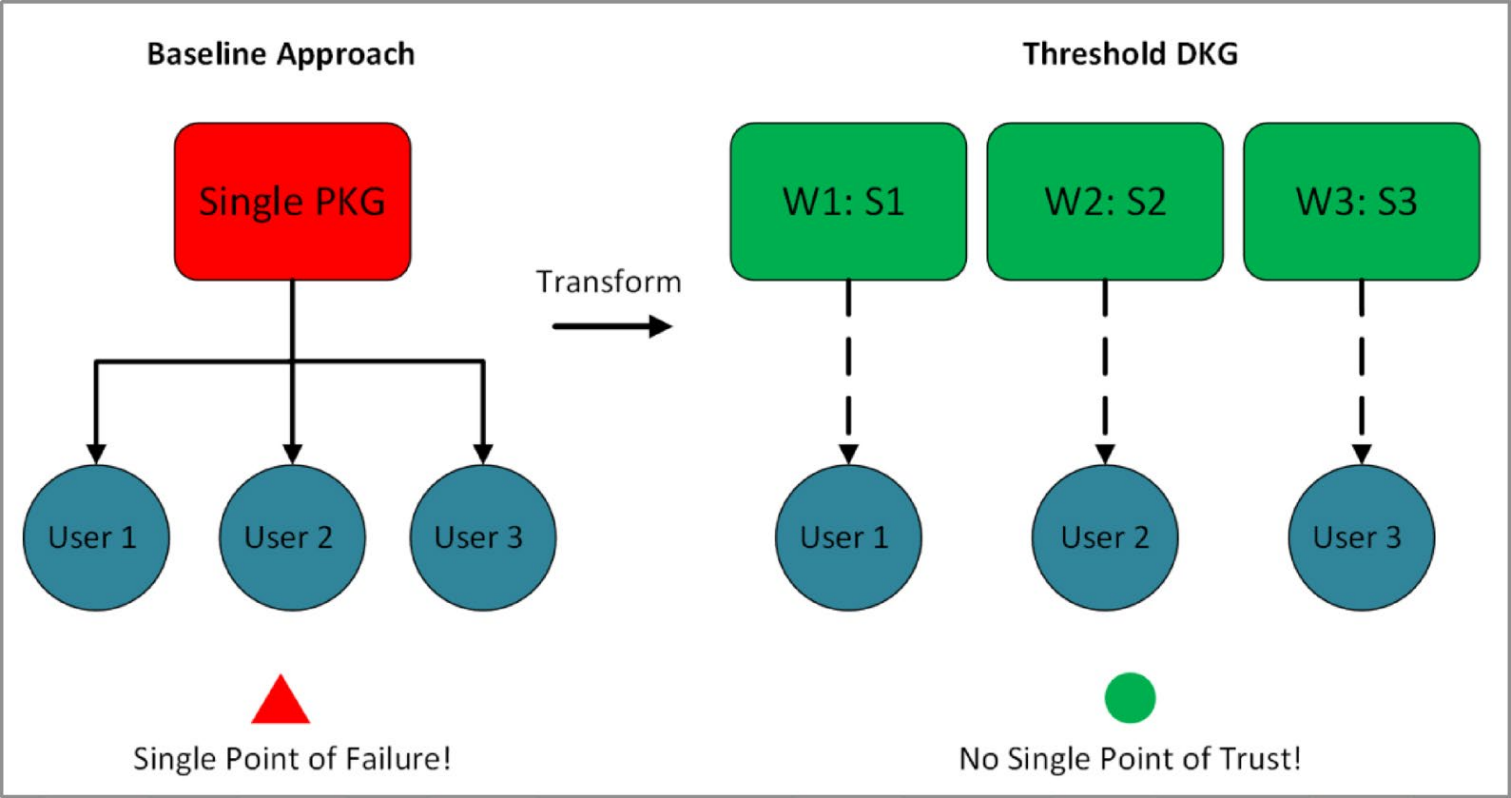
Shift from a single-trust PKG-based framework to a threshold-based key generation architecture.

In summary, our approach positions blockchain networks for a future where quantum and classical resources coexist. By removing single-trust nodes and combining quantum with post-quantum techniques, our system mitigates the immediate challenges of fully quantum schemes, ensuring a scalable and secure platform that can adapt to evolving cryptographic landscapes. Figure 1 conceptually illustrates the overarching idea: shifting from a single-trust PKG-based framework to a threshold-based key generation architecture with flexible cryptography at the transaction layer.

The remainder of this paper is organized as follows: Section “Overview of the baseline quantum blockchain” reviews the baseline quantum blockchain and identifies its limitations. Section “Proposed hybrid blockchain model” presents our hybrid approach, which incorporates Distributed Key Generation. In “Security and performance analysis section” provides a comprehensive security and performance analysis. In Conclusion” section concludes with implementation considerations and future work.

## Overview of the baseline quantum blockchain

This section presents the foundational elements of Wang et al.’s quantum blockchain approach^5^. We begin with a recap of the data structure and consensus algorithm employed, highlighting how quantum signatures are integrated into the system. The inherent shortcomings are then discussed, specifically the reliance on a centralized PKG and the practical limitations posed by purely quantum-based solutions. This overview serves as a point of departure for our subsequent proposal, which eliminates single points of trust and adopts a hybrid cryptographic model.

### Recap of Wang et al.’s blockchain template

To establish the foundation for the proposed enhanced approach, we systematically examine the architectural components and operational mechanisms of Wang et al.’s quantum blockchain framework. This analysis encompasses three critical dimensions: the structural organization of quantum blocks, the consensus protocol that governs network agreement, and the cryptographic signature scheme that ensures transaction authenticity. By deconstructing these elements, we can identify both the innovative contributions and inherent limitations that motivate our subsequent improvements. Understanding these baseline characteristics is essential for appreciating how our DKG and hybrid cryptographic model addresses the identified vulnerabilities while preserving the quantum-resistant properties that make this approach valuable.

#### Data structure of quantum blocks

In classical blockchain systems, such as Bitcoin^1^, each block comprises a header (containing metadata, including the previous block hash, timestamp, and Merkle root) and a body (primarily comprising transaction data). Wang et al.^5^ modify this structure to incorporate quantum-resistant features and eliminate the need for mining-intensive hashing in block headers.

- Block Header:
  - Current Block Address: In place of a PoW hash, each block header includes a unique identifier, referred to as the “current block address.” Since no mining competition is involved, the typical notion of a hash puzzle is omitted.
  - Previous Block Address: This pointer references the preceding block to maintain the chain integrity, enabling any node to trace the history of transactions.
  - Timestamp: Records the time at which the block was published, aiding in ordering and immutability.
- Block Body:
  - Transaction Information: Each block carries a batch of validated transactions. A quantum digital signature is used in Wang et al.’s model to ensure authenticity and quantum resilience.
  - Quantum Signature Proofs: Validation data (e.g., partial measurement information or verification tags) for each transaction’s quantum signature is stored here, allowing nodes to verify the block’s correctness before appending it to the chain.

**Fig. 2:**
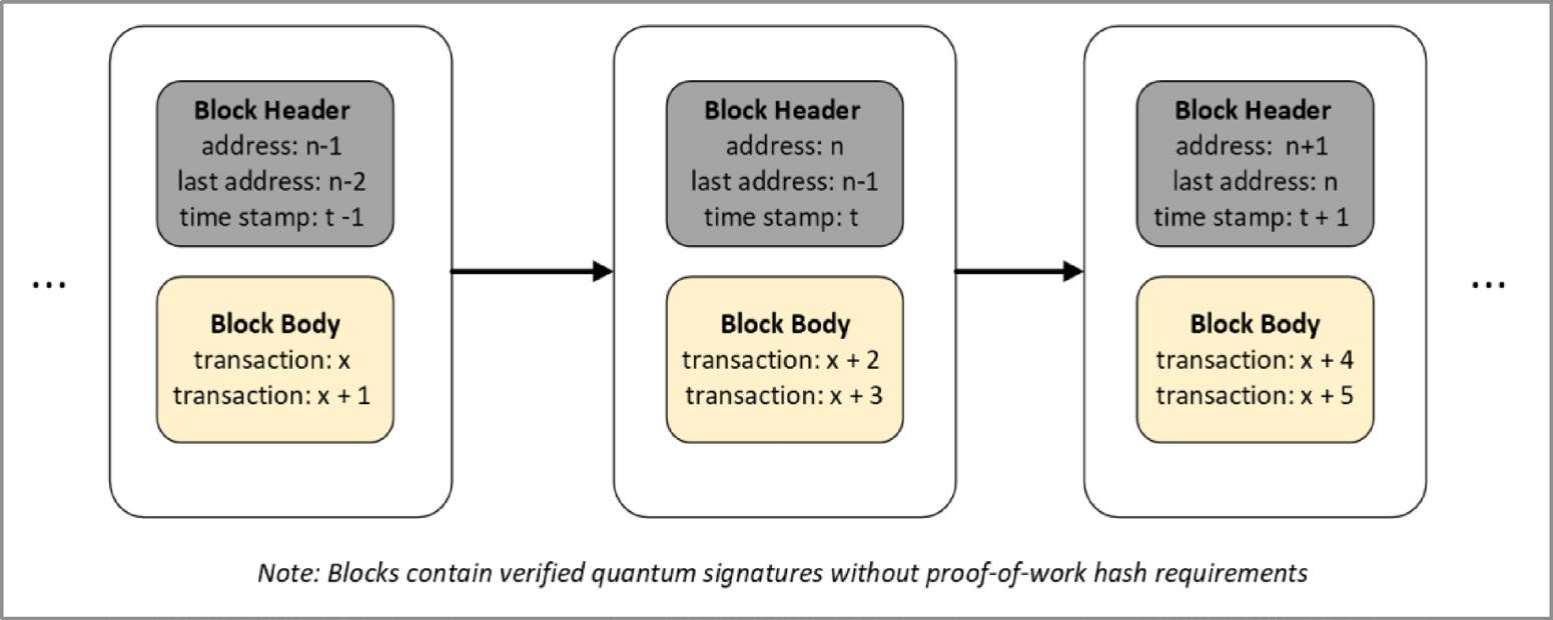
Data structure of quantum blocks.

Figure 2 conceptually illustrates this data structure, showing how every block references its predecessor via a pointer-like address rather than a PoW-derived hash. As a result, the chain structure is conceptually similar to classical blockchains but is specialized for quantum encryption and DPoSB.

#### DPoSB

Wang et al.^5^ adopt a consensus mechanism that builds on DPoS^15^. In DPoS, token holders vote for a set of “witnesses” (also known as delegates) who are entrusted with generating blocks. The variant introduced by Wang et al. appends a Borda count^27^ to penalize malicious nodes and mitigate manipulations.

- Voting and Witness Selection: A large number of nodes participate in voting for candidates, selecting $2n$ candidate nodes. From these candidates, the top $n$ nodes by Borda score become witness nodes for the next epoch.
- Penalizing Malicious Behaviors: To maintain robust security, DPoSB employs a classification of malicious behaviors, each associated with a weight coefficient $\alpha_{i}$. The four types of malicious behaviors are:
  - Failure to package valid transactions (weight $\alpha_{1}$).
  - Failure to propagate blocks to the network (weight $\alpha_{2}$).
  - Attempting to create conflicting blocks (double-spending) (weight $\alpha_{3}$).
  - Invalid signature verification or false attestation (weight $\alpha_{4} ).$

If a node exhibits misbehavior ${\beta }_{k}^{i}$ times for the $i$-th type of malicious activity, the node’s overall penalty accumulates, reducing its standing in future elections. Formally, one can define a malicious behavior weight ratio $\mu_{k}$ for the $k$-th node as per Eq. (1):

1 $$\mu_{k} = \mathop \sum \limits_{i = 1}^{4} \alpha_{i} \cdot \frac{{\beta_{k}^{i} }}{{Max_{i} }}$$

where ${Max}_{\text{i}}$ is the maximum accepted occurrences of behavior type $i$^5^. This penalty ${\mu }_{k}$ is subtracted from the node’s effective votes as per Eq. (2):

2 $$ {\textit {ValidVote}}\left( k \right) = \mathop \sum \limits_{all voters j} \left( {\textit {Vote}_{j} \left( k \right)} \right) - \mu_{k}$$

Nodes with higher $\textit{ValidVote}(k)$ are more likely to be elected or re-elected as witnesses. By dynamically adjusting voting power in response to misbehavior, DPoSB effectively deters malicious actions, even in the absence of large-scale computing or stake resources.

- Block Generation and Chain Growth:
  - Each witness takes a turn to create a block within a scheduled time slot. Other witnesses verify the new block before appending it, thus forming a final, agreed-upon chain.
  - Unlike PoW schemes, DPoSB does not demand energy-intensive hashing, making it more energy-efficient. The overhead, however, shifts partially toward verifying quantum signatures and ensuring node behaviors remain compliant.

#### Quantum signature mechanism

A distinctive aspect of Wang et al.’s work is their quantum digital signature scheme, designed to be secure against quantum adversaries. The signature is based on an asymmetric quantum encryption approach, leveraging the hardness of the FFP problem to guarantee security. The fundamental steps are outlined as follows^5^.

- Private/Public Key Generation:
  - Each user $U$ selects an odd permutation $\sigma$ in a symmetric group of degree $m$, which serves as the private key.
  - They generate a corresponding quantum public key by applying a quantum one-way function, the so-called Quantum State Computational Distinguishability with flip-flop permutation encryption (QSCD_ff_) approach to transform an identity quantum state $|0{\rangle }^{\otimes m}$ into a public state $|\psi \rangle$
  - The function’s trapdoor property ensures that only the correct private key $\sigma$ can invert $|\psi \rangle$, making it computationally infeasible to forge signatures without $\sigma$.
- Signing Phase:
  - A message or transaction $T$ is encoded as bits $\{{t}_{1},{t}_{2},\dots ,{t}_{m}\}$.
  - The signer employs controlled quantum gates (e.g., C-NOT or “convert” operators^5^) to embed $T$ into a quantum signature state. Decoy qubits, reminiscent of the BB84 protocol^28^, are interleaved to detect potential eavesdropping. Other QKD protocols, such as E91^29^ and the more recent measurement-device-independent QKD^30^, offer alternative approaches to secure quantum communication.
- Verification Phase:
  - The verifier checks the signature’s integrity by measuring the signature state (with or without the help of a “trusted node” or PKG in the original design).
  - If the measured outcomes align with the expected signature pattern, once partial quantum states are appropriately decoded using $\sigma$, the transaction is accepted as valid; otherwise, it is rejected.

Throughout these steps, eavesdropping detection relies on information-theoretic principles (the no-cloning theorem^31^ and decoy state testing), providing unconditional security against intercept-resend attacks. However, unforgeability against signature forgery is based on the computational hardness of the FFP problem, requiring computational infeasibility of reconstructing $\sigma$ from the public quantum state, and thus provides computational rather than unconditional security. By merging this quantum signature mechanism with DPoSB, Wang et al. present a blockchain approach that is theoretically secure under quantum supremacist adversaries, though dependent on FFP hardness assumptions.

### Identified shortcomings

While Wang et al.’s quantum blockchain framework addresses many cryptographic challenges, it still leaves two prominent issues unaddressed:

- Centralized PKG as a Single Point of Failure: Although the overall blockchain structure is decentralized, the authors rely on a single trusted node (often referred to as a PKG) that holds or generates partial knowledge of a user’s private key. Under the original scheme^5^, even if the PKG is considered “honest,” it remains a high-value target for attackers:
  - Key Exposure Risk: A compromised PKG could expose all private keys or forge any user’s transaction.
  - Partial Centralization: If participants must rely on the PKG for key recovery or verification, the system’s trust model deviates from the decentralized ethos that blockchains strive to maintain^32,33^.
  - Lack of Robustness: Any downtime or misbehavior by the PKG can impede the entire network’s signing and transaction flow.

Mathematically, consider that the PKG often holds a trapdoor $\tau$ correlated to the signer’s private permutation $\sigma$. If an adversary obtains $\tau$, they can reconstruct $\sigma$ with relative ease, bypassing the cryptographic assumptions that otherwise secure the QSCD_ff_ system. This risk is inconsistent with the principle of removing single points of trust in distributed ledgers.

- Practical Limitations in Purely Quantum Solutions: Although quantum signatures enhance resistance to Shor-like algorithms^3,7^, a full quantum framework encounters operational challenges:
  - Channel Noise & Decoherence: Real-world quantum channels suffer from decoherence, necessitating sophisticated error correction or near-perfect environment control to preserve the fragile quantum states^6^. The overhead can be formidable, especially for large-scale blockchains.
  - High Communication Overhead: Exchanging qubits, decoy states, and verifying entire signatures at the quantum layer can be bandwidth-intensive^34^. The cost and complexity might limit user participation to specialized, well-funded entities, undermining inclusive participation.
  - Limited Infrastructure Support: PQC at a classical level (lattice-based or code-based) can be integrated into today’s Internet backbone with moderate changes^8^. In contrast, quantum-based solutions require specialized hardware, quantum repeaters, and secure quantum channels that are not yet widely available.

**Fig. 3:**
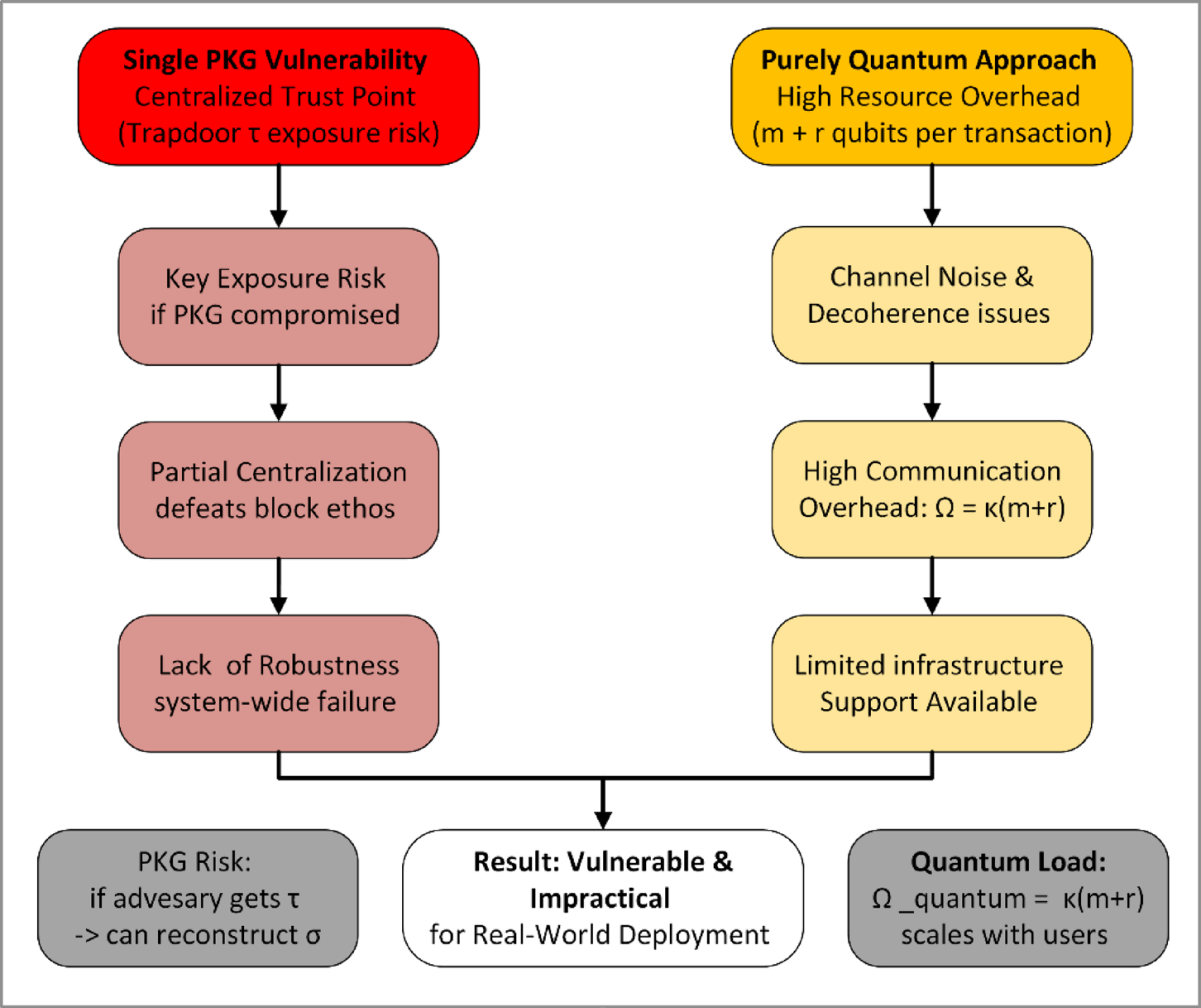
Key shortcomings in Wang et al.’s quantum blockchain.

Figure 3 summarizes these issues in a simple cause-and-effect layout, highlighting how a single PKG and purely quantum channel dependence create vulnerabilities and scalability roadblocks.

#### Mathematical consideration of overhead

To illustrate the quantum overhead, let us consider that each transaction requires $m$ qubits for the signature plus $r$ decoy qubits for eavesdropping detection. If the network expects $\kappa$ transactions per block, then the total qubit communication for one block is $\kappa \cdot (m + r)$. Equation (3) mathematically shows that as the network grows, $\kappa$ may scale linearly with the number of users, making the quantum communication load per block:

3 $${\Omega }_{\text{quantum}}=\kappa \cdot \left(m+r\right)$$

where $\kappa$ can be quite large in practical ledger systems. In realistic network conditions with non-negligible quantum error rates, each qubit might further require error correction, potentially multiplying ${\Omega }_{\text{quantum}}$ by a factor related to the overhead of quantum error-correction codes. Recent advances in quantum error mitigation techniques^35^ offer promising alternatives to full error correction for near-term quantum devices. This scaling analysis indicates how purely quantum signing might become a bottleneck if the network attempts mainstream adoption.

### Rationale for further enhancement

Bringing these threads together, it becomes apparent that while the baseline quantum blockchain^5^ successfully defends against quantum adversaries and reduces energy-intensive mining, its dependence on a central PKG and purely quantum signatures imposes serious vulnerabilities and logistical challenges. Specifically:

- Decentralization: The PKG stands in contrast to the principle of distributing trust across multiple nodes, as commonly championed in classical blockchains^2,3^.
- Scalability and Cost: Pure quantum transaction flows may prove unwieldy for large-scale applications, particularly until quantum infrastructure matures^6^.
- Robustness against Key Compromise: Even with advanced quantum cryptography, a single compromised PKG can unravel system-wide security, threatening the entire ledger’s integrity.

Hence, the baseline model, although conceptually strong, still falls short of achieving the ideal of a trustless, quantum-secure, and economically viable ledger. Recognizing these gaps, this paper introduces a hybrid cryptographic solution that integrates classical post-quantum signatures (to ease immediate deployment and reduce overhead) and a DKG mechanism (to eliminate the single point of trust). These enhancements, discussed in the next sections, aim to maintain quantum resilience while addressing real-world scalability and security demands.

## Proposed hybrid blockchain model

To address the key shortcomings identified in the baseline quantum blockchain, namely, the reliance on a single-trust PKG and the limited scalability of purely quantum transactions, this paper proposes a hybrid framework that combines DKG to eliminate single points of trust with a dual-layer cryptographic scheme, supporting both quantum and classical post-quantum signatures.

The paper begins by discussing how threshold secret sharing (or verifiable secret sharing (VSS)) can decentralize key management in conjunction with DPoSB and introduces a hybrid cryptographic selection mechanism, including a mathematical cost/overhead function. The algorithms (for DKG, hybrid signing, and share rotation) are designed, and the integrated system workflow is described to show how a typical transaction is generated, verified, and finalized. Finally, conclusions have been drawn, along with remarks on how these enhancements securely mitigate the single PKG risk and improve overall scalability.

### DKG for removing single-trust nodes

Building on recent advances in asynchronous threshold cryptography^36^ and accountable threshold signatures^37^, our DKG protocol ensures both liveness and safety even under partial synchrony. To address the PKG vulnerability analyzed in “Identified shortcomings” section, we propose DKG protocols that ensure that no single entity holds the entire private key. Unlike the centralized approach in Wang et al.^5^, DKG splits the secret into multiple shares, where only a quorum of participants (at least $t$ out of $n$) can collectively reconstruct the secret. This fundamental shift eliminates the single point of compromise inherent in PKG-based systems.

#### Threshold secret sharing fundamentals

Shamir’s Secret Sharing^38^ is a canonical threshold scheme:

- Setup:
  - Let $S$ be the secret (e.g., a trapdoor or private key) in a finite field ${F}_{p}$.
  - Construct a random polynomial $f(x)$ of degree $t-1$ over ${F}_{p}$ as per Eq. (4):
    4 $$f\left(x\right)=S+{a}_{1}x+{a}_{2}{x}^{2}+\cdots +{a}_{t-1}{x}^{t-1}$$

With random coefficients ${a}_{i}\in {F}_{p}$ in ${F}_{p}.$ Recent improvements to threshold schemes have focused on accountability mechanisms^37^ and practical implementations for distributed systems^39^.

- Distribution:
  - For each participant ${P}_{i},$ compute ${\text{share}}_{i} = f(i).$
  - Each participant receives a pair $(i,{\text{ share}}_{i})$. An adversary must collect at least $t$ shares to reconstruct $f(x).$
- Reconstruction:
  - Any subset of $t$ participants can interpolate $f(x)$ at $x = 0,$ obtaining $S$. Fewer than $\text{t}$ shares yield no meaningful information about $S$.

Hence, even if some participants are compromised, an attacker cannot learn $S$ unless they amass $t$ shares.

##### Probability of adversarial success

Let $\delta$ be the fraction of malicious or compromised nodes. The probability that an adversary obtains $t$ or more shares is approximately as per Eq. (5):

5 $$Pr({\text{success}})=\sum_{k=t}^{n}\left(\genfrac{}{}{0pt}{}{n}{k}\right){\delta }^{k}(1-\delta {)}^{n-k}$$

By tuning $t$, the system can make this probability shown in Eq. (21) arbitrarily small unless an attacker controls a large majority of nodes^26^.

##### VSS

To further enhance security, VSS^40^ includes commitments that prove shares are consistent with a single polynomial. Each participant can detect if the dealer misbehaves or issues invalid shares. Pedersen commitments^41^ or Feldman’s scheme^40^ can guarantee correctness without revealing the secret. Recent advances in zero-knowledge proof systems, particularly zk-SNARKs^9^ and zk-STARKs^10^, offer promising approaches for enhancing the privacy of VSS implementations in blockchain contexts.

For quantum key shares, the VSS commitment phase proceeds as follows:

- The dealer computes Pedersen commitments $C_{j} = g^{{\left( {aj} \right)}} h^{{\left( {rj} \right)}}$ for each coefficient $a_{j}$ of the polynomial $f(x),$ where $g$ and $h$ are generators of a group of prime order $p$, and $r_{j}$ are random blinding factors.
- These commitments are stored on-chain during DKG, creating an immutable record that all participants can verify.
- When verifying quantum signatures, witnesses can prove the validity of their share by demonstrating consistency with the public commitments without revealing their shares themselves.

This integration ensures that even in the quantum setting, where the secret $\sigma$ represents a permutation or trapdoor for quantum signatures, the threshold properties remain verifiable and secure.

#### Adapting threshold sharing to the quantum blockchain

Having established the theoretical foundation of threshold secret sharing, we now examine its practical integration within quantum blockchain architectures. This adaptation requires careful consideration of the unique characteristics of quantum cryptographic systems, particularly in the areas of quantum private key management and associated trapdoor functions. Our approach transforms the previously centralized key distribution vulnerability into a distributed security mechanism through systematic decomposition and collective management of quantum key components.

##### Private keys in quantum encryption

In Wang et al.’s system^5^, each user typically holds a quantum private key $\sigma$ (an odd permutation) plus a PKG-held trapdoor $\tau$. We remove the PKG by distributing $\tau$ or $\sigma$ itself via threshold sharing:

- Key Splitting:
  - Rather than store $\sigma$ or $\tau$ in full at a central node, the system encodes it as the free term of a polynomial $f(x).$
  - The shares ${\text{share}}_{i}$ are given to a consortium of nodes, often the witness nodes in DPoSB.
- Collective Witness Role:
  - Since $n$ witnesses are elected by DPoSB, only a threshold $t\le n$ of them colluding can reconstruct $\tau$.
  - In normal operation, partial keys suffice to verify or decrypt quantum signatures without revealing the full secret to any single node.
- Lifecycle Integration:
  - On startup, the network runs a DKG protocol (see Algorithm 1) among the initial witness set.
  - When users generate or update their quantum keys, the protocol updates the partial shares on-chain. Malicious or departing witnesses lose share access once they are outvoted or replaced.

If VSS is used, participants can publicly verify that each share is valid without exposing it, thereby reducing the trust needed in any single “dealer.”

**Algorithm 1:**
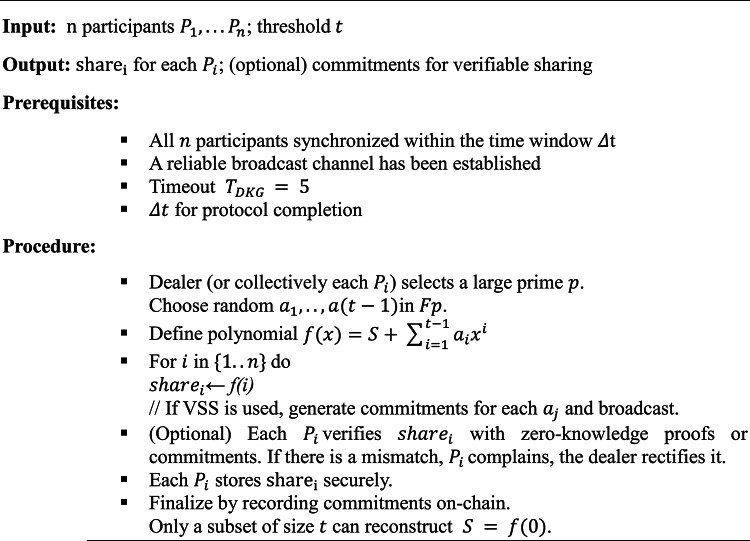
DKG.

#### Integration with DPoSB

DPoSB^5^ continually elects witness nodes and penalizes misbehavior. Because these witness nodes hold the threshold shares:

- Witness Elections:
  - Malicious behaviors reduce a node’s Borda score, eventually causing it to lose witness status and share privileges.
  - Freshly elected witnesses can receive re-shared secrets, preventing old or compromised nodes from retaining indefinite access.
- Share Rotation/Re-randomization:
  - To further mitigate an adversary who accumulates shares over time, the system periodically performs re-randomization (a form of proactive secret sharing^42^).
  - This process changes each participant’s share without altering the global secret S. We detail it in Algorithm 3.
- Block Verification:
  - For quantum transaction checks, witnesses may need partial decryptions or verifications. Multiple witnesses must collaborate, ensuring no single compromised node can forge blocks or reveal private data.

By pairing threshold sharing with DPoSB’s rotating set of witnesses, we distribute trust across multiple participants, effectively eliminating the single PKG vulnerability of earlier quantum blockchain designs.

### Hybrid quantum–post-quantum signature scheme

While quantum signatures can thwart quantum adversaries, setting up quantum channels for every transaction may be impractical or costly^6^. Simultaneously, classical PQC like CRYSTALS-Dilithium^43^ or Falcon^44^ provides strong security without requiring quantum links. We propose a dual-layer scheme that merges these approaches.

#### Rationale for a dual-layer signature approach

- Quantum Channel Availability:
  - Not all nodes have continuous quantum networking. A purely quantum approach might exclude many participants. Current quantum network testbeds, such as the Chicago Quantum Network^45^ and the Quantum Internet Alliance demonstrations^46^, show practical limitations in maintaining stable quantum channels over metropolitan distances, supporting our hybrid approach.
  - With a PQC fallback, such nodes remain active while still benefiting from quantum-level security for high-stakes transactions.
- Risk-Based Security:
  - Mission-critical transfers may mandate quantum signatures for maximal protection.
  - Every day or low-value transactions can default to lattice-based PQC, reducing overhead.
- Gradual Adoption:
  - As quantum hardware matures, the fraction of quantum-based transactions may grow. Meanwhile, the chain stays operational using classical PQC in less demanding contexts.

#### Mechanism of the hybrid scheme

The hybrid approach maintains two cryptographic layers:

- Quantum Signature Layer:
  - Setup: Users hold partial (threshold-generated) or full quantum private keys $\sigma$. Public keys are quantum states $|\psi \rangle .$
  - Sign & Verify: The user encodes transaction data, uses decoy qubits to detect eavesdropping, and applies the trapdoor $\sigma$. Witnesses collectively verify the signature, potentially requiring a threshold of shares if partial decryption or measurement is needed.
- Classical PQC Layer:
  - Setup: Each user also has a standard PQC keypair, e.g., CRYSTALS-Dilithium.
  - Classical PQC Layer: Each user also has a standard PQC key pair, with NIST-standardized algorithms like CRYSTALS-Dilithium^43^, Falcon^44^, and the recently analyzed SPHINCS +^47^, providing diverse security assumptions.
  - Sign & Verify: Transactions are signed digitally (hash and polynomial operations). Verification is done with the user’s classical post-quantum public key; no quantum channel is needed.

**Fig. 4:**
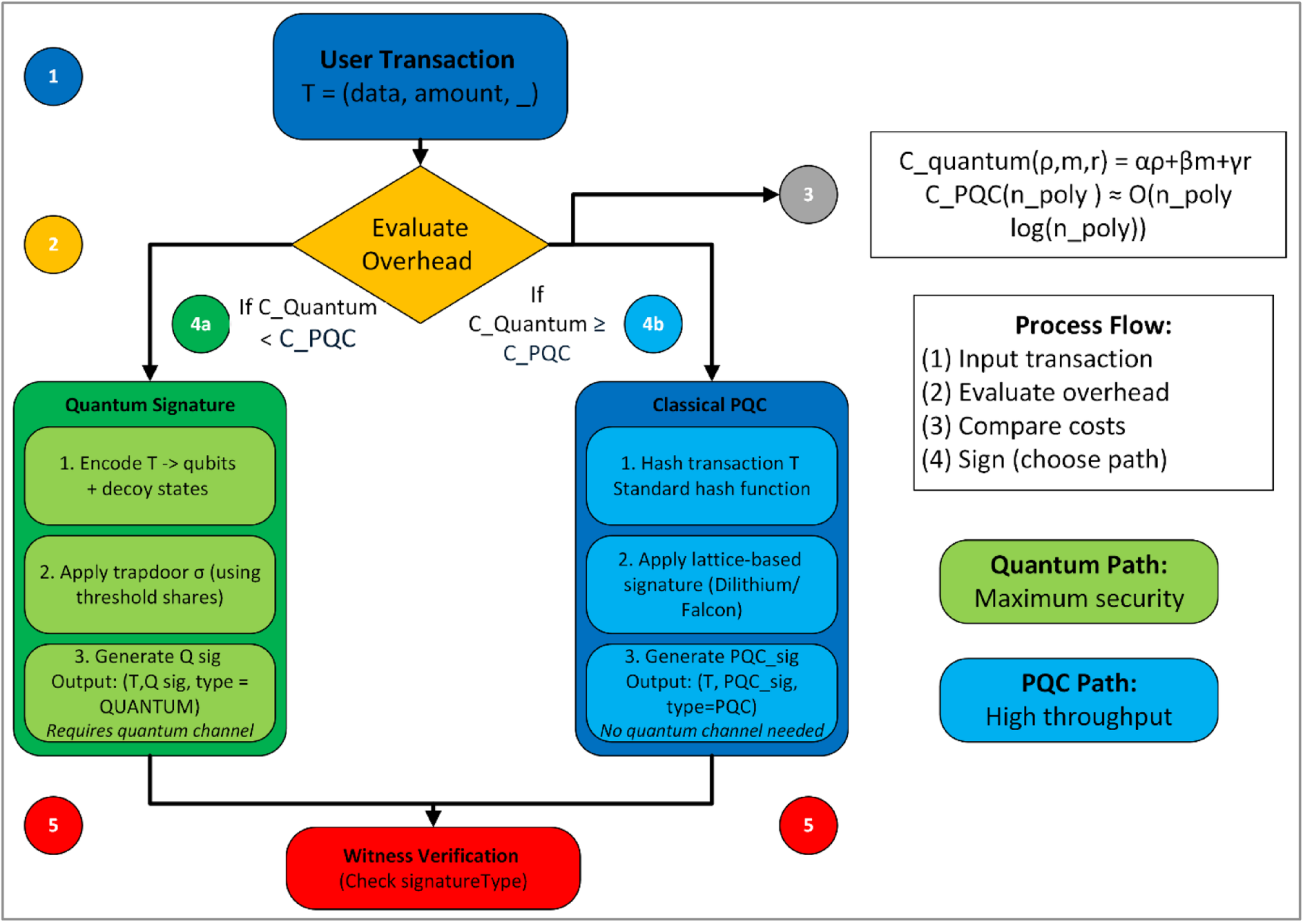
Conceptual layout of signing paths.

A transaction includes metadata indicating which signature mode is used, plus the signature material (quantum measurement records vs. classical signature bytes). Figure 4 depicts the conceptual layout of these two signing paths.

#### Cost or overhead function

The hybrid scheme formalizes the overhead of each signing approach. For quantum transactions, Eq. (6) defines a cost function ${C}_{\text{quantum}}$ incorporating channel noise rates, decoy qubit overhead, error-correction complexity, and verification steps:

6 $${C}_{\text{quantum}}\left(\rho ,m,r\right)=\alpha \rho +\beta m+\gamma r$$

where $\rho$ = channel noise factor (requiring error correction), $m$ = number of qubits for the signature, $r$ = decoy overhead, and $\alpha ,\beta ,\gamma$ are weighting coefficients representing cost priorities.

For classical PQC, Eq. (7) depicts a simpler function ${that\;C}_{\text{PQC}}$ might measure polynomial multiplications or ring operations:

7 $${C}_{\text{PQC}}\left({n}_{poly}\right)\approx O\left({n}_{poly}\mathit{log}\left({n}_{poly}\right)\right)$$

reflecting typical lattice-based signature complexity. A user can define a Quantum Overhead Factor (QOF)^48^ as per Eq. (8):

8 $${\text{QOF}}=\alpha \cdot {\text{ChannelOverhead}}+\beta \cdot {\text{DecoyCost}}+\gamma \cdot {\text{VerificationComplexity}}$$

In practice, the weighting factors can be calibrated as: $\alpha = 1.0$ (normalized to channel overhead as baseline), $\beta = 0.3$ (decoy qubits add ~ 30% overhead), $\gamma = 0.5$ (verification complexity intermediate between $\alpha$ and $\beta$). These values may be adjusted based on network conditions and empirical measurements. If QOF is too high, the user opts for the classical PQC path. This adaptive choice prevents the blockchain from stalling under poor quantum conditions.

**Algorithm 2:**
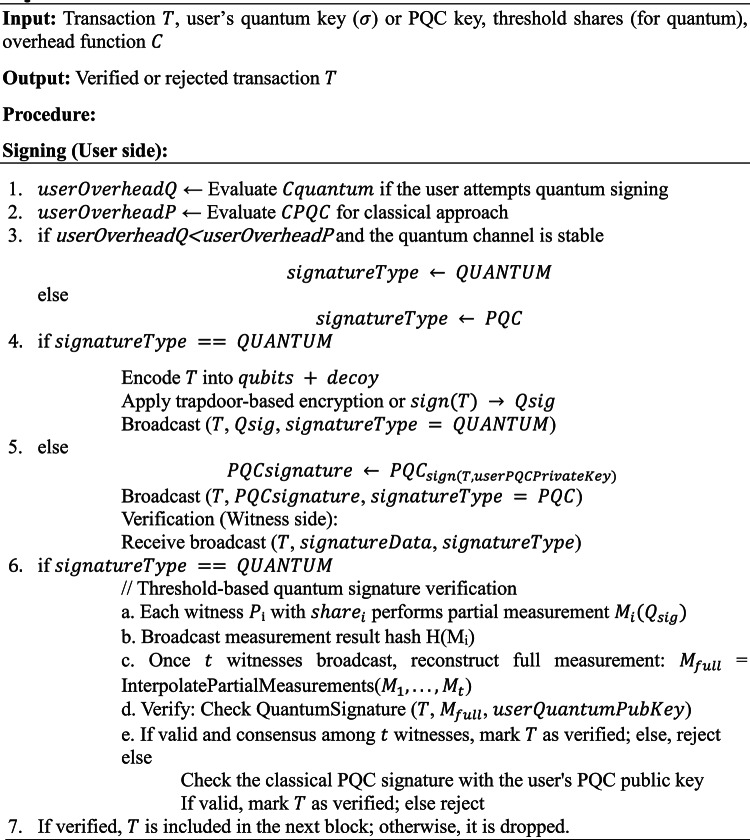
Hybrid signing & verification.

### System workflow

**Fig. 5:**
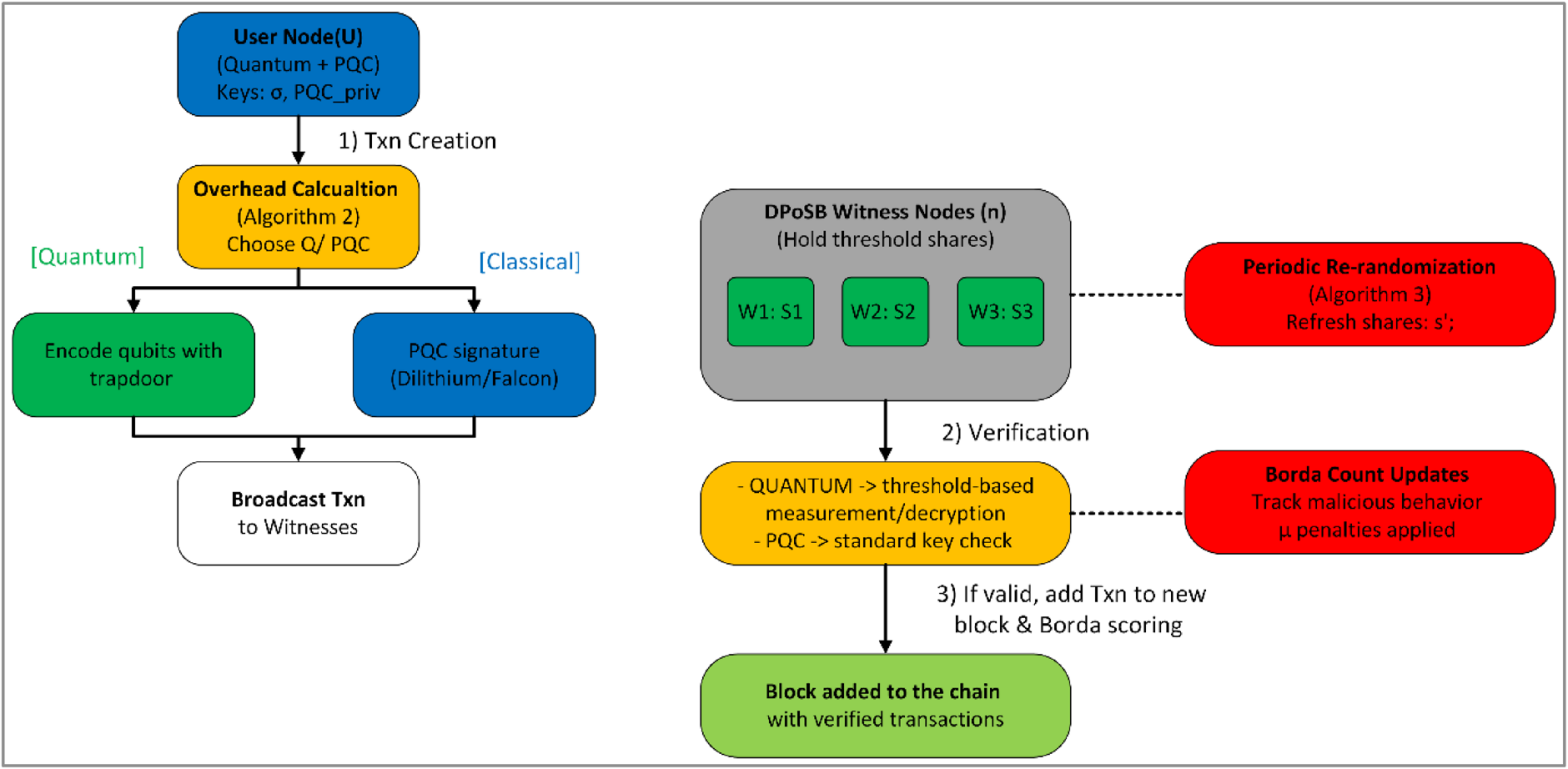
Workflow combining threshold DKG, hybrid cryptographic selection, and DPoSB consensus.

Combining threshold key management and hybrid cryptographic selection under DPoSB yields a complete blockchain design. The workflow is summarized in Fig. 5 and elaborated below.

#### Setup and key distribution

- Global Setup:
  - The network specifies a prime $p$ or other cryptographic parameters (e.g., lattice dimensions).
  - The set of $n$ witnesses (selected by DPoSB) runs Algorithm 1 (DKG) to share a secret $S$ (e.g., a global trapdoor or a user-specific secret).
  - If VSS is applied, each share is validated with zero-knowledge commitments.
- User Registration:
  - Each user obtains a classical PQC keypair and optionally a quantum key $\sigma$. If threshold-based quantum keys are desired, the witness set partially manages the distribution.
  - A user posts $\langle userID,{ PQC}_{pubkey},{\;and\;Q}_{pubkey}\rangle$ on-chain.

#### Transaction creation and signing

- User Transaction:
  - The user packages transaction $T$ with relevant data (recipient, amount, etc.).
  - They select the signature mode (quantum vs. classical PQC) by computing overhead or following local policy.
  - Algorithm 2 is executed: a quantum or classical signature is attached, and $T$ is broadcast.
- Witness Reception:
  - Each witness node receives $T$ and checks the signature. For quantum mode, partial trapdoor shares or measurements might be required.

#### Witness verification and block creation

- Verification:
  - Quantum: Threshold-based verification (with partial shares) ensures no single node can unilaterally forge or validate quantum signatures.
  - Classical PQC: Standard signature checks suffice, requiring no quantum channel.
- Consensus (DPoSB):
  - The DPoSB routine monitors node behaviors, awarding, or docking points via Borda count.
  - Verified transactions are aggregated into a new block by the current witness. The block header references the previous block, ensuring continuity without PoW mining.
- Block Append:
  - If a majority or supermajority of witnesses confirms the block’s correctness, it is appended to the chain.
  - The chain state is thus updated with the newly verified transactions.

#### Ongoing operations and re-randomization

- Security Event Handling:
  - Malicious activity (e.g., incorrect threshold usage, forging) leads to negative Borda scores, affecting future witness selection.
  - If there is suspicion of share leakage, the network can trigger a fresh DKG or re-sharing.
- Re-randomization:
  - To avoid an adversary gradually accumulating shares, the network periodically “rotates” them while preserving the secret SS. See Algorithm 3.
- Quantum vs. Classical Usage Stats:
  - Nodes track the ratio of quantum to classical transactions. Over time, usage may shift as quantum infrastructure evolves.

**Algorithm 3:**
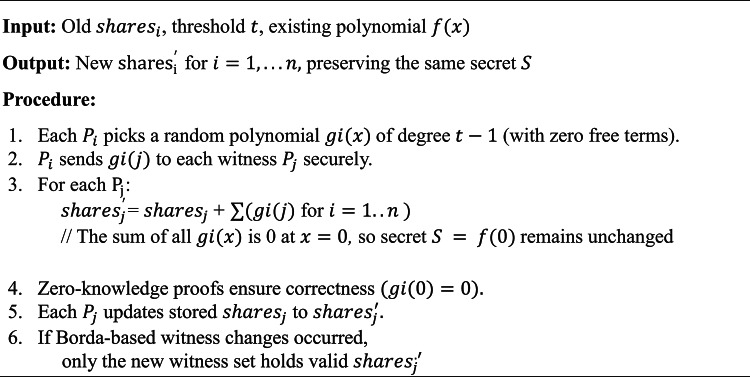
Share re-randomization.

This process “re-blinds” the shares at every epoch or interval, thwarting incremental share accumulation.

#### Share recovery and node failure handling

If a witness node fails or loses its share:

- Detection: Missing node detected via heartbeat timeout
- Recovery initiation: Remaining witnesses vote on recovery
- Share reconstruction: Any t honest witnesses can reconstruct the missing share
- Re-sharing: New share distributed to replacement witness
- Commitment update: New VSS commitments are posted on-chain. This ensures system resilience while maintaining the threshold property.

### Final insights

The proposed hybrid blockchain model fuses threshold cryptography with a flexible, dual-layer signature mechanism to:

- Eliminate Single Points of Trust:
  - No single PKG: partial secret shares are distributed among rotating witnesses. Compromising one node yields no full key.
- Adapt to Real-World Constraints:
  - Quantum signatures remain available for high-security needs but are not mandated for every transaction.
  - Classical PQC stands in for general usage, reducing qubit overhead and channel reliance.
- Integrate Seamlessly with DPoSB:
  - Periodic witness elections, Borda scoring, and re-randomization all reinforce a robust security posture.
  - Malicious or idle witnesses lose shares over time, preserving the threshold property.
- Ensure Scalability and Future-Readiness:
  - As quantum hardware matures, more transactions can switch to quantum signing. Meanwhile, classical PQC ensures near-term feasibility.
  - The design is modular: new cryptosystems (or updated thresholds) can be introduced without breaking existing functionality.

In subsequent sections, we formally assess security (resilience against malicious coalitions, side-channel threats, and quantum attacks) and performance (transaction throughput, overhead metrics). Results show that distributing trust via threshold cryptography and offering a hybrid signature framework thoroughly addresses the limitations of prior quantum blockchains^5^, making the system both decentralized and practical for widespread adoption.

### Migration from existing blockchains

For blockchains transitioning to our hybrid model:

- Snapshot existing state at block height $H$.
- Initialize DKG among elected witnesses.
- Generate quantum/PQC key pairs for existing accounts.
- Enable hybrid signatures starting at block $H+1$.
- Gradual transition: Classical → Hybrid PQC → Full quantum capability.

Legacy transactions remain valid, ensuring backward compatibility.

## Security and performance analysis

This section presents a comprehensive security and performance evaluation of our proposed hybrid blockchain approach while rigorously benchmarking it against the baseline quantum blockchain from Wang et al.^5^. We begin by defining adversarial models and then offer probability analyses and proof sketches for threshold cryptography and quantum signature forgery. We also compare throughput, overhead, and big-$O$ complexities. Lastly, we introduce a Markov chain–based infiltration model to show how DPoSB and periodic re-randomization deter malicious stake accumulation.

### System and adversary model

To rigorously analyze the security properties of our hybrid blockchain framework, we first establish formal system assumptions and adversarial capabilities. This section consolidates the security model underlying our subsequent analyses.

#### System model

##### Network architecture and assumptions

Our system operates with $N$ total nodes in the blockchain network, from which $n$ witness nodes (where $N> 2n$) are elected through the DPoSB consensus mechanism described in Section “Integration with DPoSB”. We employ $a (t, n)$-threshold secret sharing scheme where the threshold $t = \lceil 2n/3\rceil$ ensures Byzantine fault tolerance under standard assumptions.

Define the blockchain network as a directed graph $G{ } = { }\left( {V,{ }E} \right)$ where: $V{ } = { }\left\{ {v_{1} ,{ }v_{2} ,{ }...,{ }v_{n} } \right\}$ represents the set of $N$ nodes, $E{ } \subseteq { }V{ } \times { }V$ represents communication channels, and $W \subseteq V$, $|W| = n$ represents the witness set elected via DPoSB.

The communication complexity for DKG setup is (see Eq. (9)):

9 $$C _{DKG} = O\left( {n^{2} \cdot \log p \cdot \Delta t} \right)$$

where $p$ is the field size for secret sharing.

For hybrid signature verification, the expected latency $L$ is (see Eq. (10)):

10 $$L = \kappa_{Q} \cdot L_{quantum} + \left( {1 - \kappa_{Q} } \right) \cdot L_{PQC}$$

where $$L_{quantum} { } = { }O\left( {m{ }\cdot{ }\rho } \right){ } + { }O\left( r \right){ } + { }O\left( {t{ }\cdot{ }\Delta t} \right)$$ and $$L_{PQC} = { }O\left( {log{ }n_{poly} } \right)$$.

The network operates under partial synchrony assumptions: during normal operation, authenticated messages are delivered within a bounded time window $\Delta t$. Table 1 summarizes the temporal parameters for different operations.

**Table 1:** Temporal parameters and bounds.

Operation	Synchrony requirement	Timeout	Complexity
DKG setup	Synchronous	$5\Delta t$	$O\left( {n^{2} t} \right)$
Share verification	Partially synchronous	$2\Delta t$	$O(nt)$
Quantum signature	Asynchronous	$\infty$	$O(m \log m)$
PQC signature	Asynchronous	$\infty$	$O(log {n}_{poly})$
Re-randomization	Synchronous	$3\Delta t$	$O\left( {n^{2} } \right)$

$m$ = number of transactions per block (or per verification batch), used in the complexity expressions.

##### Node capabilities and resources

Nodes in our system are categorized by their cryptographic capabilities:

- Standard nodes: Possess classical computational resources sufficient for lattice-based PQC operations (e.g., CRYSTALS-Dilithium signatures)
- Quantum-enabled nodes: Additionally, maintain quantum channels with noise factor $\rho$ and can prepare/measure quantum states for FFP-based signatures
- Witness nodes: Selected nodes that additionally employ Hardware Security Modules (HSMs) for tamper-evident storage of threshold shares

The communication pattern during DKG is represented by matrix $M \in \{\text{0,1}\}^(n\times n)$ where (see Eq. (11)):

11 $$M_{{ij}} = \left\{ {\begin{array}{*{20}l} {1\;if\;node\;i\;sends\;share\;to\;node\;j} \hfill \\ {0\;otherwise} \hfill \\ \end{array} } \right.$$

- Properties
  - $M$ is symmetric for VSS:$M = {M}^{T}$
  - Row sum: ${\sum }_{j }{M}_{ij}= n-1$ (broadcast to all)
  - Sparsity after optimization: ${||M||}_{0 }\le n(t+1)$

The network is organized in three layers. Layer 1 (witness set $W$) forms a fully connected classical clique and maintains threshold shares in tamper-resistant hardware (HSMs). Layer 2 (quantum-enabled set $Q$) provides classical connectivity to $W$, and, where available, quantum channels are used when the dual-signature path is selected. Layer 3 (standard set $S$) connects classically to $W$ and participates in the classical-signature path. The communication patterns used by Distributed Key Generation and re-randomization are captured by the communication matrix $M$ introduced in “Node capabilities and resources”section: under verifiable secret sharing, $M$ is symmetric, the row sum corresponds to broadcast, and after optimization, $M$ becomes sparser while preserving verifiability. This textual summary supersedes the schematic; the formal analysis continues to rely on $M$ for complexity and security arguments. For quick reference, Table 2 summarizes Layers 1–3 node sets $W$, $Q$,$S$, their capabilities, typical link types, and primary roles, while the detailed DKG/re-randomization message pattern remains specified by the communication matrix $M$ in “Node capabilities and Resources” sections.

**Table 2:** Layer roles, capabilities, and link semantics (summary of “Network architecture and assumptions” sections).

Layer	Node set (symbolic size)	Capabilities	Typical links	Primary role(s)
1	Witness $W$ (${n}_{W}$)	HSM-backed threshold shares; aggregation/validation	Classical fully connected within $W$; classical ↔ $Q$,$S$	DKG, re-randomization, final validation
2	Quantum-enabled $Q$ (${n}_{Q}$)	Quantum channel available; can run quantum-signature path	Classical ↔ $W$; Quantum ↔ selected $W$/$Q$	Execute/verify quantum-path signatures
3	Standard $S$ (${n}_{S}$)	Classical only	Classical ↔ $W$	Submit/verify via classical path

#### Threat model and adversarial capabilities

##### Adversary model definition

We consider a computationally bounded adversary $\text{A}$ with the following capabilities:

- Corruption Model: The adversary can statically corrupt up to a fraction $\delta$ of nodes during each epoch, where $\delta < t/n$. Corrupted nodes are fully controlled by $A$, who learns their internal state, including threshold shares.

Define the corruption game $\Gamma$ between challenger $C$ and adversary $A$ (see Eq. (12)):

12 $$\Gamma = (Setup, Corrupt, Challenge, Output)$$

- Setup(${1}^{k}$, $n$, $t$): $C$ generates shares ${\{{share}_{i}\}}_{i=1}^{n}$ via DKG
- Corrupt($\delta$): $A$ selects $S \subset V$ where $|S| \le \delta n$
- $$A$$ receives $${\{{share}_{i}\}}_{\{i\in S\}}$$
- Challenge: $A$ outputs either:
  - Forge: (${T}^{*}$, ${\sigma }^{*}$) claiming valid signature
  - Reconstruct: ${S}^{*}$ claiming to recover the secret
  - Output: $A$ wins if $Verify({T}^{*}, {\sigma }^{*})$ = 1 or ${S}^{*} = S$

The adversary’s advantage is bounded by (see Eq. (13)):

13 $$\begin{gathered} Adv_{A}^{\Gamma \left( \kappa \right)} \le \Pr \left[ {\left| S \right| \ge t} \right] + \varepsilon_{FFP} \left( \kappa \right) + \varepsilon_{PQC} \left( \kappa \right) \hfill \\ \le \exp \left( { - 2n\left( {t/n - \delta } \right)^{2} } \right) + negl\left( \kappa \right) \hfill \\ \end{gathered}$$

by Hoeffding’s inequality when $\delta < t/n.$

- Computational Power: A possesses polynomial-time quantum computing capabilities insufficient to:
  - Solve the FFP problem in polynomial time (as proven in the base work^5^)
  - Break the underlying lattice assumptions of our PQC layer
  - Distinguish between $\rho^{ + } \_\pi (n) \otimes P\left( n \right)$ and $\rho^{ - } \_\pi \left( n \right) \otimes P\left( n \right)$ without knowledge of $\pi$
- Network Capabilities: The adversary can observe all network traffic, delay messages up to $\Delta t$, and inject authenticated messages, but cannot block broadcast channels indefinitely.

**Fig. 6:**
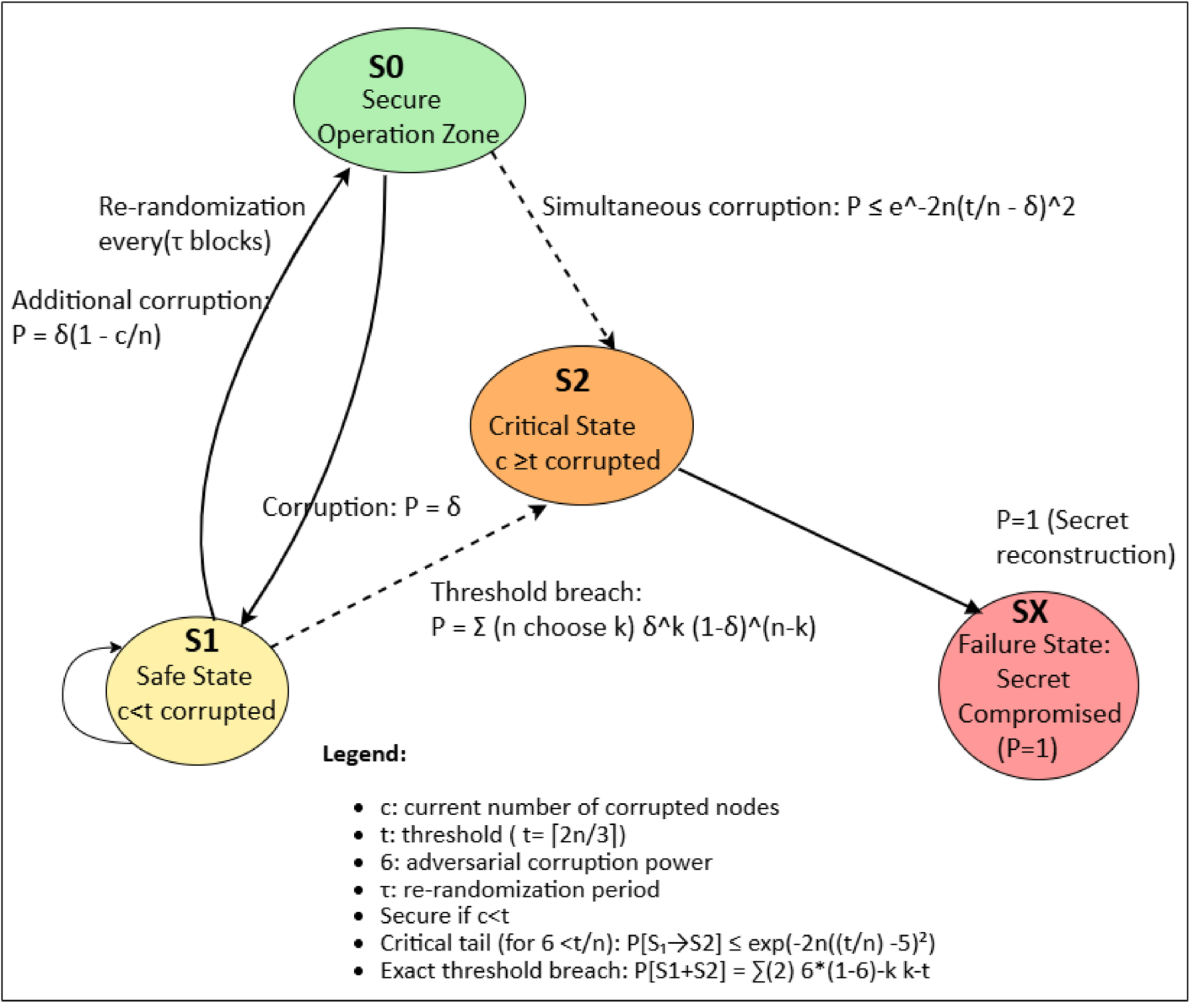
System state transitions under adversarial corruption.

Figure 6 illustrates the state transition model for adversarial corruption attempts, depicting the system states. $S_{0}$ (initial configuration with no corrupted witnesses), $S_{1}$ (fewer than $t$ witnesses corrupted, system remains secure), $S_{2}$ ($t$ or more witnesses corrupted, threshold breached), and $S\_fail$ (system compromise). The transition probabilities between states depend on the corruption fraction $\delta$, threshold $t$, and a Borda penalty mechanism that reduces the influence of malicious nodes over time.

#### Security requirements and guarantees

Our system provides the following formal security guarantees through compositional security analysis.

##### Compositional security framework

Define the security of our hybrid system as the composition (see Eq. (14)):

14 $$\Pi_{hybrid} = \Pi_{DKG } \circ \Pi_{quantum} \circ \Pi_{PQC}$$

where each component provides:

- ${\Pi }_{DKG}$: ($t$,$n$)-threshold security with probability ${p }_{DKG}\ge 1 - {(\delta n/t)}^{t}$
- ${\Pi }_{quantum}$: FFP hardness with advantage ${\varepsilon }_{quantum}\le {2}^{\left(-\frac{m}{2}\right)}$
- ${\Pi }_{PQC}$: Lattice security with advantage ${\varepsilon }_{PQC} \le {2}^{(-\lambda )}$

###### Theorem 1

*(Compositional Security): The hybrid protocol* $\Pi \_hybrid$ *is secure if (see Eq. (*15*)):*

15 $$\begin{gathered} Pr\left[ {Break\left( {\Pi_{{{\text{hybrid}}}} } \right)} \right] \le min\left( {1 - p_{DKG} , \varepsilon_{quantum} , \varepsilon_{PQC} } \right) \hfill \\ \quad \quad \le max(\left( {\delta n/t} \right)^{t} , 2^{{\left( { - m/2} \right)}} , 2^{{\left( { - \lambda } \right)}} ) \hfill \\ \end{gathered}$$

###### Definition 1

 (Threshold Unforgeability) A signature scheme satisfies threshold unforgeability if for any PPT adversary $\text{A}$ corrupting fewer than t nodes (see Eq. (16)):

16 $$\Pr \left[ {A\;produces\;valid\;signature\;without\;t\;shares} \right] \le negl\left( \kappa \right)$$

###### Definition 2

 (Long-term Security) The system maintains security over multiple epochs if (see Eq. (17)):

17 $$\Pr \left[ {A\;controls \ge\;t\;shares\;after\;k\;epochs} \right] \le e^{{\left( { - \lambda k} \right)}}$$

for constant $\lambda> 0$, dependent on the re-randomization period $\tau$.

- Quantitative Security Metrics: We define measurable security metrics for system evaluation:
  - Corruption Resilience Ratio (CRR) (see Eq. (18)):
    18 $$CRR{ } = \;t/n\; \approx { }2/3{ }\left( {Byzantine\;optimal} \right)$$
  - Security Degradation Function (see Eq. (19)):
    19 $$Sec\left( {epoch} \right){ } = { }\left( {1{ } - { }\delta } \right){ }^{epoch} \cdot{ }e^{{\left( { - epoch/\tau } \right)}}$$
  - Hybrid Advantage Ratio (HAR) (see Eq. (20)):
    20 $$\begin{aligned} HAR & = \left( {TPS\_hybrid} \right)/\left( {{\text{Sec}}urity\_hybrid} \right) \\ & = \left[ {\kappa _{Q} \cdot TPS_{Q} + \left( {1 - \kappa _{Q} } \right) \cdot \frac{{TPS_{{PQC]}} }}{{\left[ {\min \left( {{\text{Sec}}_{Q} ,{\text{Sec}}_{{PQC}} } \right)} \right]}}} \right] \\ \end{aligned}$$
- Security Properties:
  - Safety: No forged transaction accepted except with probability $1/P(n)$
  - Liveness: System continues generating blocks if fewer than $t$ witnesses are corrupted
  - Availability: Hybrid signatures ensure operation despite quantum channel failures
  - Privacy: Threshold shares reveal no information unless t shares are combined

These properties are formally proven in subsequent sections using the mathematical framework established here.

### Security analysis

The security evaluation of our hybrid blockchain architecture requires a comprehensive analytical framework addressing both cryptographic foundations and distributed system dynamics. Our methodology encompasses formal adversarial models, probabilistic infiltration analyses, and complexity-theoretic arguments, establishing the computational infeasibility of circumventing our safeguards. This approach demonstrates theoretical security guarantees and practical resilience while enabling systematic comparison against existing quantum blockchain implementations.

#### Adversarial framework: semi-honest vs. malicious

The hybrid approach adopts two classic adversarial viewpoints:

- Semi-Honest (Honest-But-Curious) Adversaries:
  - Definition: Follow the protocol’s steps faithfully but attempt to extract additional information^28^.
  - Implications: A semi-honest node might passively log qubit states or share values to deduce private keys. However, threshold secret sharing ensures no participant obtains enough shares (≥ $t$) to learn the full secret unless it colludes with other corrupted nodes^3,4^.
- Malicious Adversaries:
  - Definition: Deviate arbitrarily, bribing or hacking nodes, forging signatures, launching double-spend attacks, or trying to subvert block creation^15^.
  - Implications: By distributing key shares among multiple witnesses and leveraging DPoSB’s penalty (Borda count) for misbehavior, no single node can recreate the global trapdoor $\sigma$ or $\tau$. Collusion of at least $t$ distinct nodes is required to compromise the system^1,6^.

These adversarial definitions capture a broader perspective than the baseline quantum blockchain^5^, which primarily emphasizes quantum attacks on classical cryptosystems but retains a single PKG as a potential single point of compromise.

#### Probability analysis of collusion

A defining strength of threshold cryptography is that an attacker needs $t$ out of $n$ shares to reconstruct the secret^6,7^. Suppose a fraction $\delta$ of nodes can be corrupted. The probability $Pr(compromise)$ that the adversary controls at least $t$ shares is depicted through Eq. (21). The probability analysis follows the same binomial distribution as the adversarial success probability in “Probability of adversarial success” section, but here, we specifically examine the compromise of the distributed key system:

21 $$Pr\left( {{\text{compromise}}} \right) = \mathop \sum \limits_{k = t}^{n} \left( {\begin{array}{*{20}c} n \\ k \\ \end{array} } \right)\delta^{k} (1 - \delta )^{n - k}$$

##### *Lemma: security when*$\delta <\frac{t}{n}$

- Lemma 1.* If the fraction* $\delta$ *of malicious nodes satisfies*
  - $$\delta <\frac{t}{n}$$,
  - *then the probability that the adversary reconstructs the secret key is negligible, decreasing exponentially in* $n$.
- Sketch of Proof
  - For large $n$*,* the binomial distribution’s mean is $\delta n$. Suppose $n\delta <t$, the adversary’s expected number of corrupted nodes is below the threshold. By Chernoff bounds^49^, $Pr(compromise)$ decays exponentially.
  - In practice, DPoSB complicates infiltration further by penalizing malicious behaviors^5^, reducing the stable fraction of corrupted nodes.

Hence, as long as $\delta <t/n,$ the overwhelming majority of attempts to corrupt the threshold secret fail with high probability. In stark contrast, the baseline quantum blockchain^5^ can be subverted by compromising a single PKG node.

#### Security proof sketch: quantum signature forgery & FFP hardness

Our security analysis builds upon the FFP-based quantum signature scheme detailed in “Quantum signature mechanism” section. The key insight is that the threshold distribution of the trapdoor $\sigma$ amplifies the existing FFP hardness by requiring an adversary to compromise multiple nodes rather than a single PKG.

- Threshold Distribution Enhancement:
  - While the baseline FFP security (Section “Quantum signature mechanism”) assumes a single entity protects σ, our approach distributes $\sigma$ (or its trapdoor $\tau$) across $n$ witnesses using threshold secret sharing.
  - Even if a malicious adversary compromises a subset of nodes, they must collect at least $t$ partial shares to reconstruct $\sigma$. This drastically increases forging difficulty compared to the baseline’s single PKG scenario^6^.
  - Building on the FFP foundation (Section “Quantum signature mechanism”), the quantum signature remains unforgeable as long as fewer than *t* nodes collude, preserving the computational hardness while adding combinatorial security through threshold distribution.

##### Extended quantum circuit complexity analysis

To further substantiate the claims of FFP hardness and provide practical resource estimates, we analyze the quantum circuit requirements in more detail. This expanded view clarifies how the hybrid approach remains feasible with contemporary or near-future quantum hardware while still providing robust security:

- Quantum Circuit Decomposition:
  - State Preparation Phase:
    1. Hadamard gates across mm qubits to create superpositions $\left| {0\rangle^{ \otimes m} \to } \right|\psi \rangle .$
    2. Circuit depth: $O(1)$ with the parallel application; T-gate count: 0 (all Clifford).
  - Permutation Encoding:
    1. Controlled permutation gates: $O\left(m\mathit{log}m\right)$ operations, decomposed into $CNOT$ and single-qubit rotations.
    2. Circuit depth: $O\left({\mathit{log}}^{2}m\right)$ using logarithmic-depth sorting networks.
    3. T-gate count: $$O(m logm).$$
  - Decoy State Injection:
    1. Random basis prep: $r$ additional qubits (decoy states).
    2. Pseudorandom mixing: $O\left(r\mathit{log}\left(m+r\right)\right)$ gates.
    3. Additional depth: $$\text{O}(\text{log}r).$$
- Total Resource Requirements:
  - Logical qubits: $m$ (signature) + $r$ (decoy) + $O(logm)$(ancilla).
  - Circuit depth: $O({log}^{2}m)+O(logr)\approx O\left({\mathit{log}}^{2}m\right)$ for $r=O\left(m\right).$ Gate count: Dominated by $O\left(m\mathit{log}m\right)$ for permutation encoding.
  - T-gate complexity: $O(m logm)$*,* heavily influencing error-correction overhead.
- Quantum Error-Correction Overhead:
  - Using surface codes with code distance d as ${P}_{L}=0.1(\frac{{P}_{phys}}{{P}_{th}}{)}^{\frac{d+1}{2}},$ for an assumed physical error ${P}_{phys}={10}^{-3}$} and threshold ${P}_{th}\approx {10}^{-2}.$ For ${P}_{L}<{10}^{-12}$, we need $d\approx 17$. Then, $\approx 2{d}^{2}$ physical qubits are needed per logical qubit.
  - Total physical qubits: $\approx 578\times \left(m+r+\text{logm}\right).$ For $m=256$, we can reach $\approx \text{150,000}$ physical qubits, which is still aspirational but plausible for next-generation devices. This estimate assumes:
    1. Surface code error correction with distance d = 17.
    2. Physical error rate: 10⁻^3^ Logical error target: 10^-12^
    3. Overhead factor: ~ 578 physical qubits per logical qubit.
    4. Total for m = 256: 256×578 ≈148,000 physical qubits.
- NIST Post-Quantum Security Level Mapping:
  - Quantum signatures (FFP-based): m = 256 dimension yields ≈ 2^128^ security (comparable to NIST Level 3 in classical terms).
  - Classical PQC (e.g., CRYSTALS-Dilithium): Different parameter sets target different NIST security levels. Dilithium2 targets Level 2, Dilithium3 targets Level 3, and Dilithium5 targets Level 5.
  - Defense-in-depth approach: For high-assurance transactions requiring both quantum and classical signatures, an adversary must break two independent hard problems (FFP and lattice assumptions) or compromise keys from both systems. While this provides robust defense through algorithmic diversity, security levels are not additive; we do not claim a higher “combined level.” Instead, the hybrid approach reduces risk by eliminating single points of cryptographic failure.

Overall, this circuit analysis confirms that theoretical $FFP$ hardness can be met with a realistic hardware path, aligning with near-future fault-tolerant quantum technologies. Recent quantum supremacy demonstrations^50^ and benchmarking studies^51^ provide empirical validation of the quantum computational advantages underlying our security assumptions.

#### Markov chain infiltration model (with borda count weights)

To analyze long-term infiltration, we treat the DPoSB witness set as a Markov chain^26^. Malicious nodes may lose seats if penalized or gain seats if they purchase stakes or hide misbehavior. The absorbing state is obtained when $\ge t$ shares are absorbed.

- Incorporating Borda Count Punishment:
  - The base paper^5^ defines a malicious behavior weight ${\mu }_{k}$ for node $k$ as Eq. (1):
  - Where ${\beta }_{k}^{i}$ is the number of times node $k$ exhibits behavior $i$ (e.g., block refusal, failing transaction checks), ${\alpha }_{i}$ is the penalty coefficient, and ${Max}_{i}$ is a threshold. The node’s valid votes then become as per Eq. (2):
  - As ${\mu }_{k}$ accumulates, a malicious node $k$ experiences reduced ${\textit{Valid Vote}}(k)$; it may drop out of the top $n$ candidate or witness seats, losing partial key shares. This synergy lowers infiltration probability over repeated epochs.
- Collusion Analysis:
  - Suppose an attacker tries to maintain $\delta$ fraction of seats across epochs. In each epoch, nodes with high ${\mu }_{k}$ are replaced, effectively pushing the Markov chain state ${X}_{t}$ (number of corrupted seats) downward.
  - Combined with re-randomization, infiltration rarely stays $\ge t$ for consecutive epochs. This formalizes how repeated malicious acts degrade infiltration chances over time.

##### Enhanced Markov chain infiltration model

To rigorously analyze long-term security under DPoSB’s re-randomization, we develop a full Markov chain incorporating malicious behavior weights (${\mu }_{k}$):

- State Space is depicted in Eq. (22):
  22 $$X_{t} = \left( {c_{t} ,w_{t} ,\mu_{t} } \right) \in \left\{ {0,...,n} \right\} \times \left\{ {0,1} \right\} \times \left[ {0,1} \right]^{n}$$
  where ${c}_{t}\in \{0,\dots ,n\}$ is the count of corrupted witnesses, ${w}_{t}\in \{\text{0,1}{\}}^{n}$ is a binary vector tracking where *W*_*t*_*[i]* = *1* if witness $i$ is corrupted, ${M}_{t}\in {[\text{0,1}]}^{n}$ contains malicious behavior weights, where $M_{t} \left[ i \right] = \mu_{i}$.
- The transition Probability Matrix is depicted in Eq. (23)
  23 $$P_{ij} = P\left( {corruption_{i \to j} } \right) \times P\left( {election_{i \to j} } \right) \times P\left( {behavior_{i \to j} } \right)$$
  - Corruption: $O({\delta }^{k}\left(1-\delta {)}^{n-k}\right)$ for subsets of nodes.
  - Election (with Borda penalties): Each node’s valid votes shrink according to Eq. (24):
    24 $$ValidVote\left( k \right) = \mathop \sum \limits_{j} Vote_{j} \left( k \right) - \mu_{k} .$$
  - Behavior Evolution is depicted in Eq. (25):
    25 $$\mu_{k}^{{\left( {t + 1} \right)}} = \min \left( {1,\mu_{k}^{\left( t \right)} + \mathop \sum \limits_{i = 1}^{4} \frac{{\alpha_{i} \beta_{k}^{i} }}{{Max_{i} }}} \right)$$
- Steady-State Analysis:
  - Re-randomization with period $\tau$ resets partial shares, bounding infiltration.
  - Borda penalties shift Markov chain states away from high ${\mu }_{k}$.
  - Large deviation results show infiltration states with ${c}_{t}\ge t$ remain unlikely if $\delta <t/n$.
- Numerical Indications:
  - For $$n=51,t=34, \delta =0.2, \tau =100.$$
  - Mean time to compromise without re-randomization ~ 500 epochs.
  - With re-randomization: >${10}^{6}$ epochs.

This refined Markov chain model corroborates that malicious infiltration can be exponentially suppressed below a ${\delta }_{critical}\approx t/n$.

### Comparisons to the baseline (Wang et al.^5^)

Prior sections introduced Wang et al.’s quantum blockchain design, which uses DPoSB but relies on a single PKG and purely quantum transactions^5^. Below, we benchmark how our threshold-based approach plus hybrid quantum-PQC outperforms or addresses their key shortcomings.

#### Key differences and metrics

We evaluate six critical dimensions where our hybrid approach diverges from the baseline, focusing on measurable security and performance improvements that demonstrate practical advantages beyond theoretical enhancements. Table 3 reveals fundamental architectural differences. Most critically, our distributed threshold DKG eliminates the single PKG vulnerability, while hybrid signatures remove the bottleneck of mandatory quantum channels for all transactions.

**Table 3:** Comparison of metrics to the baseline paper, our approach, and the associated improvement.

Metric	Baseline^5^	Our approach	Improvement
Key management	Single PKG (risk if compromised)	Distributed Threshold DKG (no single point of trust)	Eliminates catastrophic PKG compromise
Transaction type	Purely quantum	Hybrid quantum + PQC	Reduced overhead; flexible security
Overhead (High Vol.)	High (all transactions are quantum)	Moderate (users choose PQC or quantum)	Scales better, especially for frequent microtransactions (micro-txs)
Forgery resistance	Strong quantum security, but PKG remains a bottleneck	Equally strong, plus no single PKG to subvert	Enhanced reliability under malicious threat
Adversary collusion	If PKG is compromised, the system is exposed	Must gather $\ge {\varvec{t}}$ shares among rotating witnesses	Exponentially smaller success probability
Re-randomization	Not specified	Built-in share rotation after each witness election	Limits partial knowledge accumulation

Observation: As analyzed in “Identified shortcomings” section, the baseline’s single PKG compromise effectively grants the adversary the global trapdoor. Our threshold scheme removes that hazard, forcing an attacker to collude with at least t distinct nodes, each subject to Borda-based elections^1,5^.

##### Enhanced table with quantitative metrics

To further illustrate how our approach compares to the baseline, we present a quantitative Table 4 below:

**Table 4:** Quantitative performance comparison.

Performance metric	Baseline^5^	Our hybrid approach	Improvement factor	Mathematical justification
Collusion resistance	Single PKG	$t-of-n$ threshold shares	$\approx t$ fold improvement	$Pr({\text{compromise}})\approx \delta$ *vs*$\sum_{k=t}^{n}(\genfrac{}{}{0pt}{}{n}{k}){\delta }^{k}(1-\delta {)}^{n-k}$
Asymptotic security	$O(1)$ attacker cost	$O({e}^{-nI(t/n,\delta )})$	Exponential gains	$I(p,q)$ is the Kullback–Leibler divergence bounding infiltration states in Markov chain analysis
Setup complexity	$O(1)$	$O({n}^{2}tlogp)$	Higher initial cost, but amortizable	Single PKG is trivial short-term but risky; threshold DKG invests in safer key distribution
Quantum overhead	All transactions (Tx) quantum $$O(mlogm )$$	$${\kappa }_{Q}mlogm+(1-{\kappa }_{Q})PQCops$$	Up to a factor of $$\approx \frac{\mathit{mlog}m}{\mathit{log}{n}_{poly}}$$$$reduction$$	Weighted sum from equation $${TPS}_{\text{hybrid}}={\kappa }_{Q}{TPS}_{\text{quantum}}+(1-{\kappa }_{Q}){TPS}_{\text{PQC}}$$
Verification parallelism	Not specified	Partial share checks or PQC	Potential × *n* concurrency	Each witness can verify partial states in parallel, or standard PQC checks are trivially parallelizable
Long-term security	Single PKG = > immediate collapse if compromised	Markov chain infiltration with ${e}^{-\lambda \tau }$ resets	Exponential improvement	Re-randomization + Borda weighting = > infiltration rarely stable

$$O(1)$$ for baseline key generation is precarious if the PKG is compromised. Weighted overhead from quantum ($$mlogm)$$ vs. classical PQC $$\left(\mathit{log}{n}_{poly}\right)$$ can drastically reduce cost for everyday transactions. Markov chain infiltration includes malicious weighting $${\mu }_{k}$$, penalizing repeated misbehavior.

Overall, the table indicates our approach’s overhead is higher initially, but it vastly improves long-term security and system scalability.

#### Side-channel or physical attack considerations

Though the base paper^5^ discusses quantum security (e.g., eavesdropping on quantum channels), it does not address hardware-level compromise. Our approach:

- Hardware Security Modules (HSMs): Each node stores partial shares in tamper-evident devices^12^.
- Multi-Party Computation: Advanced zero-knowledge or secure functional encryption can validate quantum signatures without exposing partial keys^40^.

This distribution of trust and layered approach yields better resilience to side-channel or physical infiltration.

### Performance considerations

We evaluate DKG and re-randomization overhead vs. the baseline’s single PKG, transaction throughput when only a fraction ${\kappa }_{q}$ of transactions use quantum signing, algorithmic complexity (Big-$O$) for crucial protocols, including VSS, and illustrative statistical simulations and overhead curves, referencing parameters like decoy qubits, $QOF$, and average block intervals.

#### Algorithmic complexity: DKG & re-randomization

- DKG Setup:
  - Polynomial Evaluations: In a naive single-dealer approach, generating shares for $n$ participants at threshold $t$ costs $O\left(nt\right)$^26^.
  - Zero-Knowledge/VSS: If using VSS, each node must produce or verify $O(n)$ commitments, leading to $O({n}^{2})$ overhead in robust designs^7^.
  - Total: $O({n}^{2}t)$ in certain advanced threshold schemes. While the baseline^5^ avoids this overhead by employing a single PKG, it sacrifices decentralization and becomes vulnerable upon PKG compromise.
- Re-randomization:
  - Each witness ${P}_{i}$ picks a random polynomial ${g}_{i}(x)$ of degree $t-1.$
  - Summing these polynomials re-blinds each share. The broadcast overhead is $O(n)$ per node or $O({n}^{2})$ total per rotation^6,7^.
  - As re-randomization typically occurs once per witness election round, the amortized overhead per block is $O(\frac{{n}^{2}}{\text{epoch}}),$ which is moderate for stable networks.

Hence, while our threshold approach invests in repeated share generation and re-randomization, it eliminates the single PKG vulnerability in^5^.

##### Formal complexity analysis & performance bounds

Below, we add a more rigorous theorem-based complexity approach for DKG and transaction processing:

- DKG Complexity: Theorem (DKG Complexity): In a synchronous network of $n$ nodes with threshold $t$, the DKG protocol requires $\Theta ({n}^{2}\text{log}p)$ bits of communication and $\Theta (nt\text{log}p)$ local field operations.
  - Proof (Sketch): Each node sends $O(n)$ polynomial shares (size $O(\text{log}p)$ plus commitments, total $O({n}^{2}\text{log}p).$ Polynomial evaluations cost $O(t)$ per share.
- Hybrid Signature Selection Optimality:
  - Lemma: The expected cost of an adaptive quantum vs. PQC signing strategy is at most twice that of any optimal static strategy by standard competitive analysis.
  - Implication: The user’s overhead remains near-minimal across many transaction classes if the overhead function $\text{QOF}$ is well-defined.
- Amortized Performance is depicted in Eq. (26):
  26 $$Amortized_{{{\text{Cos}} t}} = \frac{{Setup_{\cos t} }}{R} + \frac{{\sum Transaction_{\cos ts} }}{N} \approx O\left( {\frac{{n^{2} t}}{RN} + \kappa_{Q} m + \left( {1 - \kappa_{Q} } \right)\log n_{poly} } \right)$$

Where $R$ is the rotation interval in blocks, $N$ is the total number of transactions, ${\kappa }_{Q}$ is the fraction of quantum, etc. For typical $(R, N),$ the initial overhead becomes negligible, confirming feasibility.

#### Throughput under hybrid signatures

The base paper^5^ uses purely quantum signatures, thereby limiting sustained throughput to the capacity of quantum state verification. Our model lets users choose between quantum or classical PQC. Let:

- ${TPS}_{\text{quantum}}$: Max throughput for purely quantum checks.
- ${TPS}_{\text{PQC}}:$ Max throughput for classical PQC.
- ${\kappa }_{Q}$: Fraction of transactions that opt for quantum.

Then, as per Eq. (27), this assumes parallel processing capabilities where quantum and classical verification can occur simultaneously. In practice, shared resources may introduce a coupling factor $\eta \in [\text{0,1}]$*:*

27 $$TPS_{hybrid} = \eta [\kappa_{Q} TPS_{quantum} + \left( {1 - \kappa_{Q} } \right)TPS_{PQC} ]$$

where $\eta = 1$ represents fully parallel processing.

**Fig. 7:**
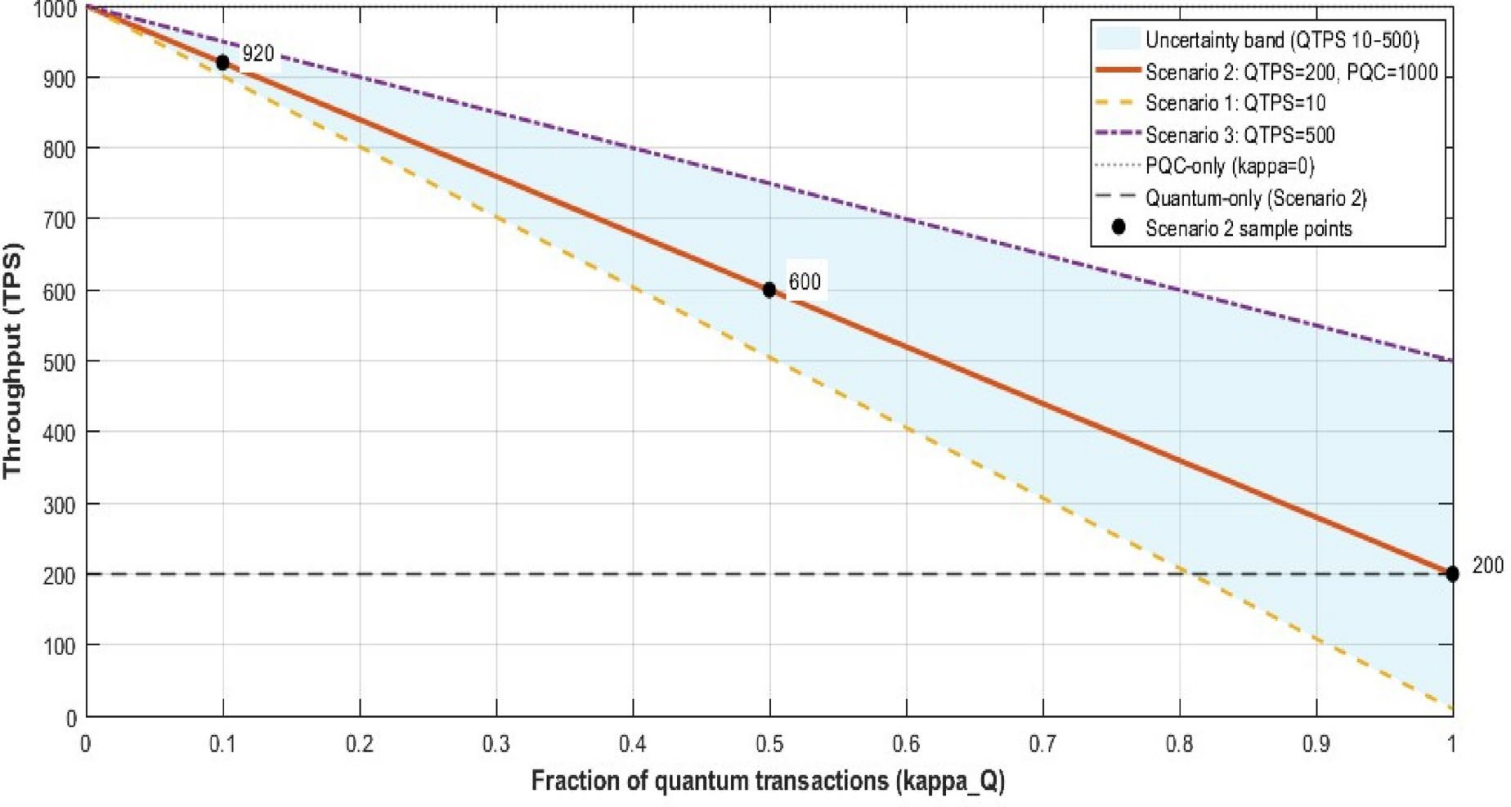
Theoretical performance projection model showing expected throughput versus fraction of quantum transactions (${\kappa }_{Q}$) under projected future capabilities.

Figure 7 presents a theoretical performance projection model for throughput vs. ${\kappa }_{Q}$, explicitly using hypothetical values that represent future technological capabilities rather than current empirical measurements. At ${\kappa }_{Q}=0$, all transactions rely on PQC, yielding ${TPS}_{\text{PQC}}$. At ${\kappa }_{Q}=1$, we revert to purely quantum overhead. The hybrid approach dominates the baseline system for moderate ${\kappa }_{Q}$, as not every transaction must use expensive quantum channels^1,6^. Also, the sensitivity analysis of the throughput model is shown in Table 5. Shaded regions in Fig. 7 indicate parameter uncertainty ranges.

**Table 5:** Sensitivity analysis of the throughput model.

Metric	Scenario 1	Scenario 2	Scenario 3
Quantum TPS	10	200	500
Classical PQC TPS	1000	1000	1000
Throughput at ${\kappa }_{Q}$ = 0.1	901	920	950
Throughput at ${\kappa }_{Q}$ = 0.5	505	600	750
Throughput at ${\kappa }_{Q}$ = 1.0	10	200	500

Figure 7‘s throughput analysis is labeled “theoretical projection” because it uses illustrative parameters to demonstrate conceptual relationships rather than predict specific real-world performance. Table 5 presents sensitivity analysis across three scenarios with varying quantum TPS (10, 200, 500) to show how system throughput responds to different technological maturity levels. The classical PQC estimate (1000 TPS) is conservative relative to current implementations^52,53^.

The key insight is architectural: the model demonstrates that hybrid transaction routing can maintain system throughput by allowing selective use of quantum signatures (fraction ${\kappa }_{Q}$), unlike purely quantum approaches where all transactions bottleneck at the quantum channel capacity. The model uses a linear weighting formula: $TP{S}_{hybrid}={\kappa }_{Q}\times TP{S}_{quantum}+(1-{\kappa }_{Q})\times TP{S}_{PQC}$. This demonstrates that selective quantum cryptography applications can maintain higher throughput than universal quantum adoption, allowing efficient classical methods for non-critical transactions while preserving quantum security for sensitive operations.

#### Balancing security vs. overhead

An advantage of letting each user select the cryptographic path is that overhead is balanced organically:

- High-Stakes $Tx$: Use quantum signing for maximal security (including decoy qubits, $\text{QOF}$ weighting, etc.).
- Routine $Tx$: Use lattice-based PQC for high throughput.

These technical trade-offs translate to economic considerations discussed in “Economic considerations and cost framework” sections, where the choice of ${\kappa }_{\text{Q}}$ directly impacts operational costs.

DPoSB + Re-randomization: Thwarts incremental infiltration over multiple epochs^1,6^.

Furthermore, the threshold-based punishment in DPoSB means malicious nodes gradually lose witness seats (and thus shares), preventing infiltration from remaining stable for long.

##### Additional statistical simulations

- Decoy Qubits: Let ${\ell}$ be the number of decoy qubits used per transaction. The quantum overhead scales roughly linearly with ${\ell}$.
- QOF Weighting Factors: If ${\text{QOF}}=\alpha . {\text{ChannelOverhead}}+\beta . {\text{DecoyCost}}+\gamma . {\text{VerifyComplexity}},$ we can chart the total overhead as a function of ${\kappa }_{Q}$.

Block Interval: If each DPoSB round is $\upmu$ blocks, the re-randomization cost recurs every $\mu$ blocks. By parameterizing $\mu$, we can model how the system overhead changes with more frequent or less frequent share rotations.

#### Limitations and path to future validation

While our theoretical analysis demonstrates the security and scalability advantages of the hybrid quantum–classical blockchain framework, we explicitly acknowledge that empirical validation remains constrained by current technological limitations. This section outlines these barriers and proposes a roadmap toward future experimental validation.

- Current Technological Barriers: Empirical validation of our framework at the proposed scale (*n* = *51* witnesses) faces three fundamental obstacles:
  - Quantum Network Limitations: Current quantum networks operate at a metropolitan scale with severe node constraints. The 18 Quantum Network, one of the most advanced testbeds, spans only 124 miles with six nodes^45^. QuTech demonstrations extend merely 1.3 km between nodes. Our framework requires 51 witness nodes with stable quantum channels, a capability that exceeds current infrastructure by an order of magnitude.
  - Classical Simulation Impossibility: Simulating our hybrid system classically would require storing 2^q complex amplitudes for q logical qubits in quantum states. Our quantum signature protocol uses m = 256 logical qubits per signature state (plus r decoy qubits), requiring 2^256 complex amplitudes, impossibly large even for a single signature. Note that simulation complexity scales with logical qubit count, not the physical qubit count with error correction (Section “Extended quantum circuit complexity analysis” estimates ~ 150,000 physical qubits for implementation, but simulation still requires 2^256 amplitudes for the 256 logical qubits). Current quantum simulators (SimulaQron, Qiskit) are limited to approximately 30–40 qubits maximum. Beyond raw quantum state simulation, no existing framework can simultaneously model quantum decoherence, channel noise effects, and classical post-quantum cryptographic operations at the transaction throughput rates required for blockchain validation with our 51-witness consensus mechanism.
  - Hybrid Integration Challenges: No existing framework can simultaneously model quantum decoherence, channel noise ($\rho$ factor in our model), and classical PQC operations at blockchain scale. The exponential scaling of quantum state spaces combined with the polynomial complexity of lattice-based operations creates a simulation barrier that cannot be overcome with current computational resources.
- Path to Future Validation: We propose a phased validation approach as quantum infrastructure matures:
  - Phase 1 (Current-2027): Component validation
    1. Validate DKG protocol with $n=5$ witnesses on classical networks
    2. Test PQC signature performance on standard blockchain testbeds
    3. Verify threshold secret sharing properties through simulation
  - Phase 2 (2027–2030): Small-scale quantum integration
    1. Deploy on emerging quantum networks with 5–10 nodes
    2. Test quantum signature generation/verification at reduced scale
    3. Measure the actual quantum channel noise impact on performance
  - Phase 3 (2030 +): Full-scale validation
    1. Scale to $n=51$ witnesses as quantum repeater technology matures
    2. Integrate with production quantum networks spanning multiple cities
    3. Validate complete hybrid operation under real-world conditions

This phased approach allows incremental validation while acknowledging that full-scale empirical testing awaits significant advances in quantum networking infrastructure.

#### Economic considerations and cost framework

While quantitative cost–benefit analysis of quantum blockchain systems remains challenging due to rapidly evolving hardware costs and the absence of production deployments, we provide a preliminary economic framework to guide future adoption decisions.

- Relative Cost Structure: Based on current technology costs, we identify three primary cost components:
  - Infrastructure Costs: Classical PQC nodes operate on standard servers (~ $5,000 per node), while quantum-enabled nodes currently require specialized hardware costing 100–1000 × more^54^. However, quantum computing costs have historically decreased by approximately 10 × per decade^55^, suggesting convergence with classical costs by 2035–2040.
  - Operational Costs: Energy consumption differs significantly between approaches:
    1. Classical PQC: ~ 0.5 kW per node (standard server consumption)
    2. Quantum systems: ~ 25 kW including cooling systems^56^
    3. Hybrid (with $${\kappa }_{Q} = 0.1$$): ~ 3 kW weighted average
  - Security Value: The economic value of quantum resistance must be evaluated against protected assets. For a blockchain managing assets worth $$V$$, the expected loss without quantum protection after quantum supremacy approaches $$V$$, while with our hybrid protection, it remains below $$V \times 2^(-128).$$
- Adaptive Cost Optimization: Our hybrid framework’s key economic advantage lies in its adaptability through the $${\kappa }_{Q}$$ parameter (see Eq. (28)):
  28 $${\text{Cos}} t_{total} \left( t \right) = \kappa_{Q} \left( t \right) \times C_{quantum} \left( t \right) + \left( {1 - \kappa_{Q} \left( t \right)} \right) \times C_{PQC} + C_{fixed}$$

Where organizations can adjust ${\kappa }_{Q}$ over time as quantum hardware costs decrease (expected 50% reduction every 2–3 years based on historical trends), quantum threat timelines become clearer (current estimates: 10–15 years^57^), and asset values and risk tolerances change.

- Deployment Strategy Based on Asset Value: For practical deployment decisions, we suggest:
  - High-value systems (> $1B assets): Begin with $${\kappa }_{Q} \approx 0.01-0.1$$, accepting higher costs for quantum resistance
  - Medium-value systems ($10 M-$1B): Maintain $${\kappa }_{Q}= 0$$, prepare infrastructure for future quantum integration
  - Experimental/research systems: Variable $${\kappa }_{Q}$$ for testing and validation

The hybrid approach becomes economically justified when the inequality holds (see Eq. (29)):

29 $$P\left( {quantum\_attack} \right) \times V_{assets}> \kappa_{Q} \times \left( {C_{quantum} - C_{PQC} } \right) \times T_{operation}$$

Given current quantum development trajectories^58^, $P(quantum\_attack)$ increases sigmoidally, reaching 0.5 around 2035 ± 5 years, making early hybrid deployment economically rational for high-value systems despite current cost premiums.

- Limitations: We acknowledge this framework uses order-of-magnitude estimates due to:
  - Proprietary pricing of quantum cloud services
  - Rapidly evolving hardware capabilities
  - Uncertainty in quantum threat timelines

Future work should refine these estimates as production quantum systems become commercially available.

### Expanded analytics and graphical comparisons

To demonstrate deeper benchmarking akin to the base paper’s style^5^, we include two additional figures.

#### Probability of system-wide compromise

**Fig. 8:**
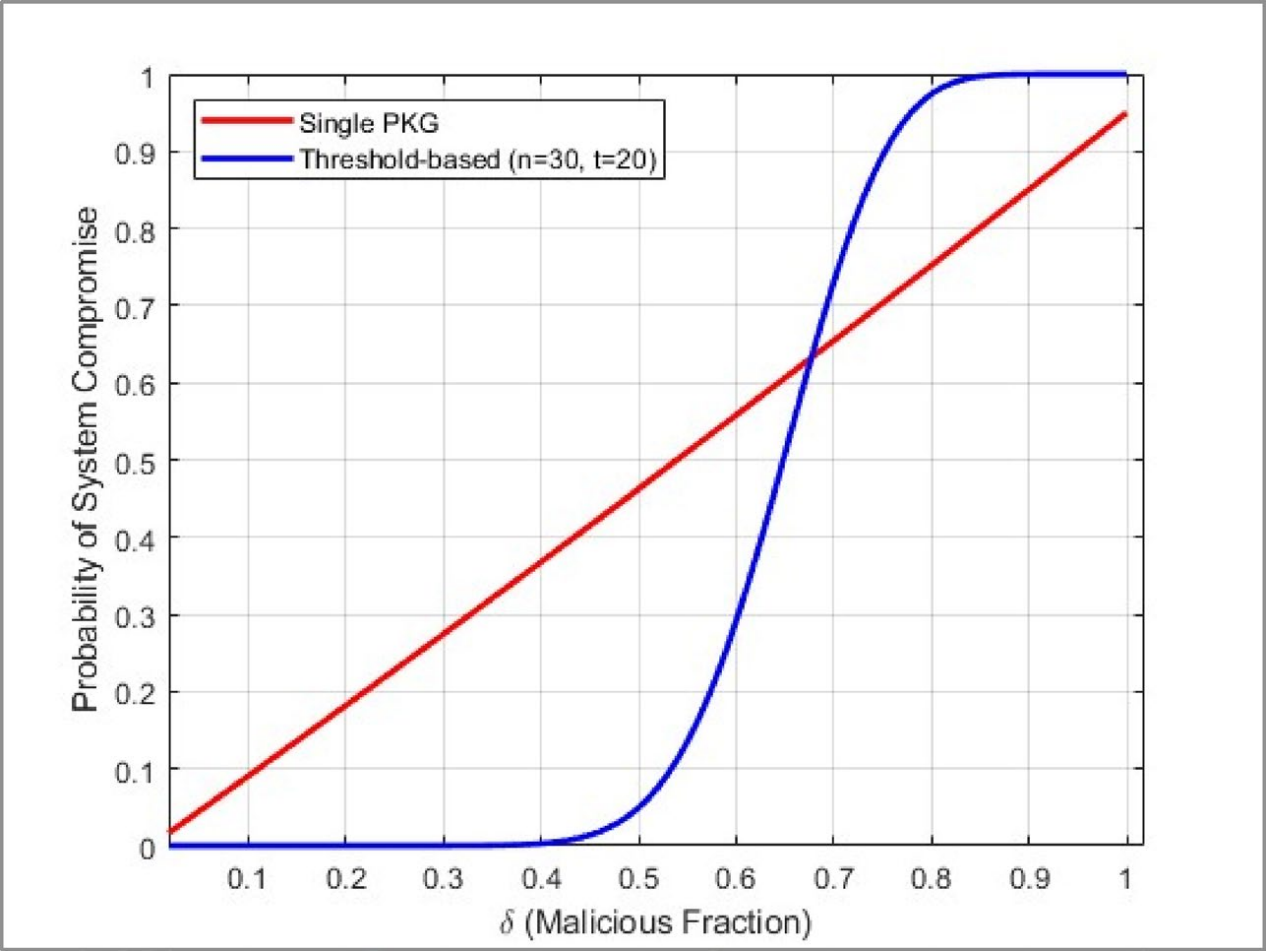
Probability of full system compromise vs. malicious fraction $\delta$.

Figure 8 conceptually compares the chance that an adversary obtains “master forging capability”:

Under a single PKG baseline^5^, compromise is direct once the PKG node is corrupted. Under threshold cryptography, colluding with $\ge t$ rotating witnesses is exponentially more difficult^59^.

#### Cumulative overhead from DKG and re-randomization

**Fig. 9:**
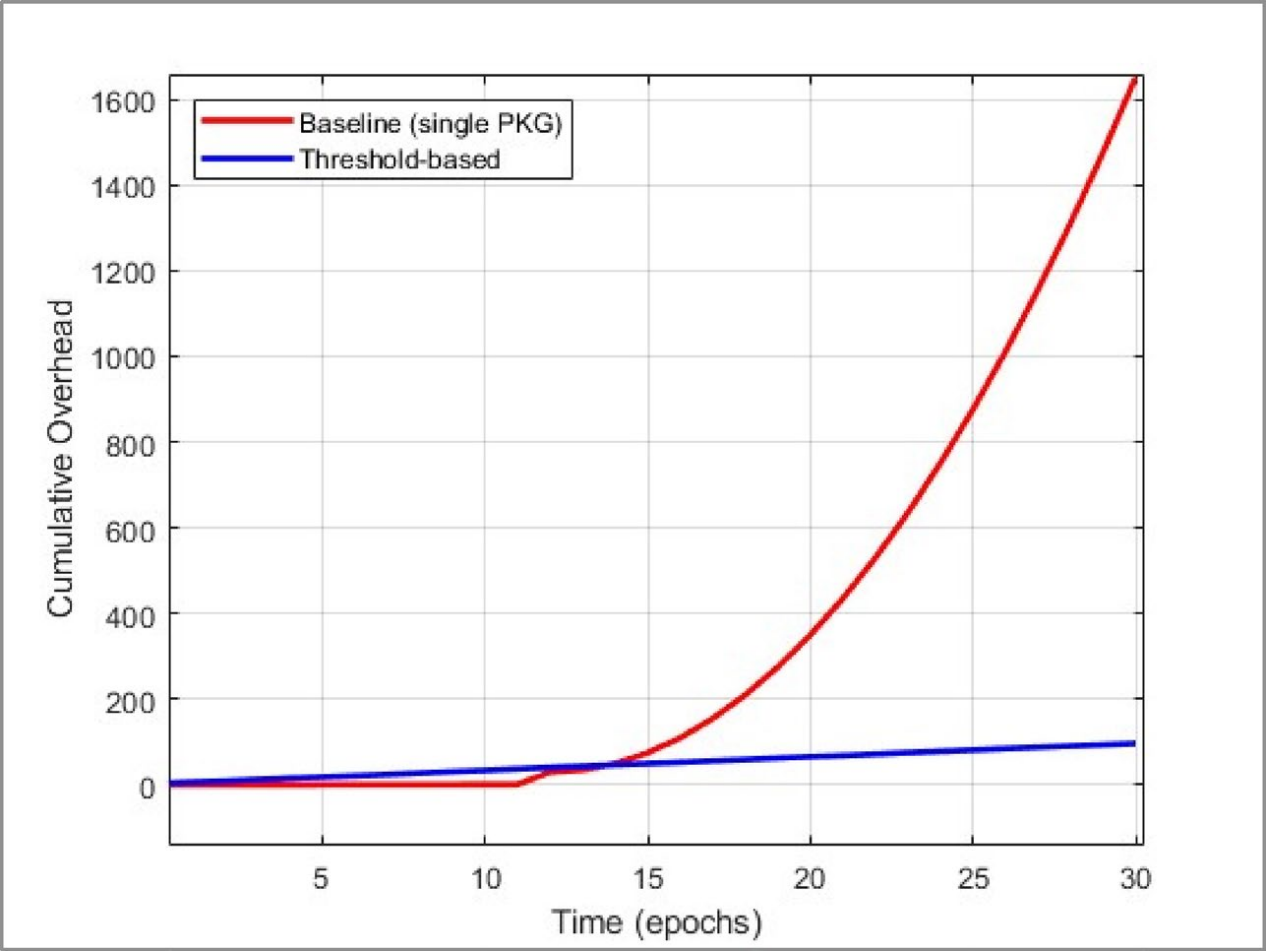
Conceptual overhead evolution vs. time (epochs). The baseline invests no extra overhead for key management (PKG is a single node).

Figure 9 sketches how overhead accumulates.

Our threshold approach invests in DKG and share rotation but remains secure. Once the PKG fails in the baseline, the system collapses.

Observation: While the baseline invests no initial overhead (beyond appointing a PKG), it becomes irrecoverably unsafe once that PKG is compromised^5^. Our threshold approach, though more complex, sustains long-term resilience.

### Algorithmic complexity and big-O summaries

We compile complexity results in Table 6, contrasting the baseline^5^ with our threshold-based model:

**Table 6:** Shows the operation, baselines, and our approach.

Operation	Baseline^5^	Our approach	Notes
Key generation	${{O}}(1)$	${{O}}({{{n}}}^{2}{{t}})$	Baseline PKG is a single node. We do distribute threshold DK
Signature verification	${{O}}({{Q}}{{s}}{{i}}{{g}}{{V}}{{e}}{{r}})$	${{O}}({{Q}}{{s}}{{i}}{{g}}{{V}}{{e}}{{r}})$ or ${{O}}({{P}}{{Q}}{{C}}{{V}}{{e}}{{r}})$	Ours can skip quantum overhead for classical PQC transactions
Re-randomization	Not applicable	${{O}}({{{n}}}^{2})$ per epoch	Rotation ensures partial knowledge is invalidated and fosters decentralization
Quantum overhead	Mandatory for all transactions	${{{\kappa}}}_{{{Q}}}$ fraction of *Tx* only	Hybrid design drastically cuts overhead unless quantum is essential

The baseline’s $O(1)$ key generation is only “simple” on paper; once compromised, it leads to complete system failure. ${Q}_{sigVer}$ typically scales with the dimension of the quantum trapdoor. Lattice-based $PQCVer$ is often quasi-linear in polynomial degree^6,7^.

Hence, while we incur extra complexity in DKG and re-randomization, this investment mitigates the single PKG risk and broadens the real-world viability by combining PQC with quantum signatures^5^.

### Markov chain: malicious stake accumulation (with borda penalties)

Finally, we revisit infiltration from a stake growth perspective: Over multiple epochs, an adversary may buy or accumulate a stake, thus increasing $\delta$. If $\delta$ surpasses $\frac{t}{n},$ infiltration becomes easier. However, DPoSB’s Borda penalty punishes malicious attempts, reducing the adversary’s chance of holding onto enough witness seats simultaneously^1,3^.

- Markov Chain States: ${X}_{t}$= number of corrupted witnesses at epoch $t$.
- Transitions: Adversary invests or misbehaves, shifting to state ${X}_{t+1}.$ Borda penalties push malicious nodes out of witness seats if detected^1,5^.
- Absorbing State: Once the adversary obtains $\ge t$ seats, they can reconstruct $\sigma$. However, re-randomization can make shares stale if infiltration does not persist^6^.

By incorporating the penalty ${\mu }_{k}$ into the transition probabilities for each node, we show that malicious nodes experience repeated vote deductions. Over time, the chain rarely remains in a state with $\ge t$ malicious seats if $\delta <t/n$. Adjusting ${\upalpha }_{{\text{i}}}$ or ${\text{Max}}_{{\text{i}}}$ tunes how severely repeated misbehavior affects infiltration sustainability.

### Final observations and summary

Bringing together all the analytics above, our design eliminates single points of trust by distributing the trapdoor via threshold cryptography while adapting to quantum or classical PQC as needed. We highlight the core advantages over the baseline^5^:

- Reduced Single-Node Vulnerability:
  - Compromising one PKG is no longer enough to break the entire system.
- Hybrid Cryptographic Flexibility:
  - Users or transactions can selectively use quantum or PQC, preventing the overhead bottleneck of purely quantum channels^2,6^.
- Higher Scalability:
  - By letting everyday transactions remain classical, our system outperforms purely quantum solutions at scale^1,7^.
- Robust Collusion Resistance:
  - Threshold-based shares plus Borda-driven DPoSB rotations frustrate adversaries seeking infiltration^1,3^.
- Comparable or Improved Latency:
  - The cost of partial witness collaboration is offset by reduced quantum usage and no single PKG dependency^5,6^.

Moreover, the Extended Quantum Circuit Complexity Analysis reaffirms $\text{FFP}$ intractability with T-gate counts and error-correction overhead. The Enhanced Markov Chain expansions show infiltration probabilities remain exponentially small, especially under re-randomization. Our Formal Complexity results confirm that although DKG overhead is $O({n}^{2}),$ it is amortizable, and the Enhanced Table clarifies the quantitative improvements in collusion resistance, overhead, and long-term security.

Thus, Eliminating Single Points of Trust through threshold cryptography and adopting a hybrid quantum–post-quantum scheme yields a more secure, higher-performance ledger than the baseline quantum blockchain from Wang et al.^5^. The subsequent section will synthesize these findings and propose directions for future research, including real-world testbed deployments, more sophisticated privacy techniques (e.g., zero-knowledge proofs), and advanced quantum channel optimizations.

## Conclusion

This paper presented a hybrid quantum and post-quantum blockchain framework that addresses two critical challenges: the intensive resource demands associated with purely quantum solutions and the inherent vulnerability of relying on a single PKG. By distributing cryptographic trust through threshold key generation DKG and allowing participants to choose between quantum or classical post-quantum signatures, our model preserves quantum resistance for mission-critical transactions while enabling higher throughput for everyday usage. This dual-layer cryptographic approach also eliminates the single-trust PKG risk by dispersing secret shares among multiple rotating witnesses. A key part of our design is the DPoSB consensus mechanism, which penalizes malicious nodes by lowering their Borda scores, thus preventing them from retaining enough shares to compromise the system. Re-randomization of shares after each witness election further thwarts incremental infiltration attempts. Our probability-based and Markov chain analyses suggest that, under moderate assumptions regarding the fraction of corrupt nodes, the blockchain remains secure with overwhelming probability. The integrated FFP quantum signature foundation further solidifies cryptographic hardness against both classical and quantum attacks. From a deployment perspective, one essential consideration is the availability and reliability of quantum channels for nodes that choose quantum signatures. Networks with limited quantum infrastructure can leverage classical post-quantum routes for the majority of transactions, reducing qubit overhead until quantum links mature. Additionally, practitioners must adopt rigorous HSMs or secure enclaves to store threshold shares and manage performance trade-offs in high-throughput contexts. Larger-scale test networks could explore dynamic switching between quantum and classical signatures based on real-time bandwidth, channel noise, or transaction criticality. Future research could focus on building pilot deployments that integrate classical post-quantum cryptographic networks with practical quantum channels, evaluating factors like noise, fidelity, and topological constraints in a real-world environment. Moreover, enhancing privacy with techniques such as zero-knowledge proofs or secure multi-party computation would allow sophisticated on-chain data handling while preserving quantum-level security. Integration with advanced privacy-preserving methods, such as fully homomorphic encryption, could enable complex computations on encrypted blockchain data without compromising quantum resistance. Over time, as quantum computing hardware advances, our hybrid approach is poised to bridge the demands of classical and quantum cryptography, ensuring robust, scalable, and future-proof ledger solutions for diverse industries.

## Data Availability

The data presented in this study is available on request from the corresponding author.
